# Supplementary material for: ZBP1 specifically binds pre-formed Z-RNA, limiting its potential pool of nucleic acid ligands compared to ADAR1
Source: Nucleic Acids Res. 2026 Oct 13;54(19):gkag972. doi: 10.1093/nar/gkag972 (PMC13659236; doi:10.1093/nar/gkag972)
Supplement: Supplementary file 1 [file gkag972_supplemental_file.pdf]

Supporting Information For:

ZBP1 Specifically Binds Pre-Formed Z-RNA, Limiting its Potential Pool of Nucleic Acid Ligands Compared to ADAR1

Jeffrey B. Krall[1], Lily G. Beck[1], Parker J. Nichols[1,2], Robb Welty[1], Quentin Vicens[3], Morkos A. Henen[1], and Beat Vögeli[1,4]*

1. Department of Biochemistry and Molecular Genetics, University of Colorado Anschutz Medical Campus, Aurora, Colorado, 80045, USA.
2. Department of Biochemistry, University of Utah School of Medicine, Salt Lake City, Utah, 84112, USA.
3. Department of Biology and Biochemistry, Center for Nuclear Receptors and Cell Signaling, University of Houston, Houston, Texas, 77204, USA.
4. RNA Bioscience Initiative, University of Colorado Anschutz Medical Campus, Aurora, Colorado, 80045, USA.

*Correspondence: beat.vogeli@cuanschutz.edu; Tel.: +1 (303) 724-1627 (B.V.)

Supporting Information & Supplementary Figures

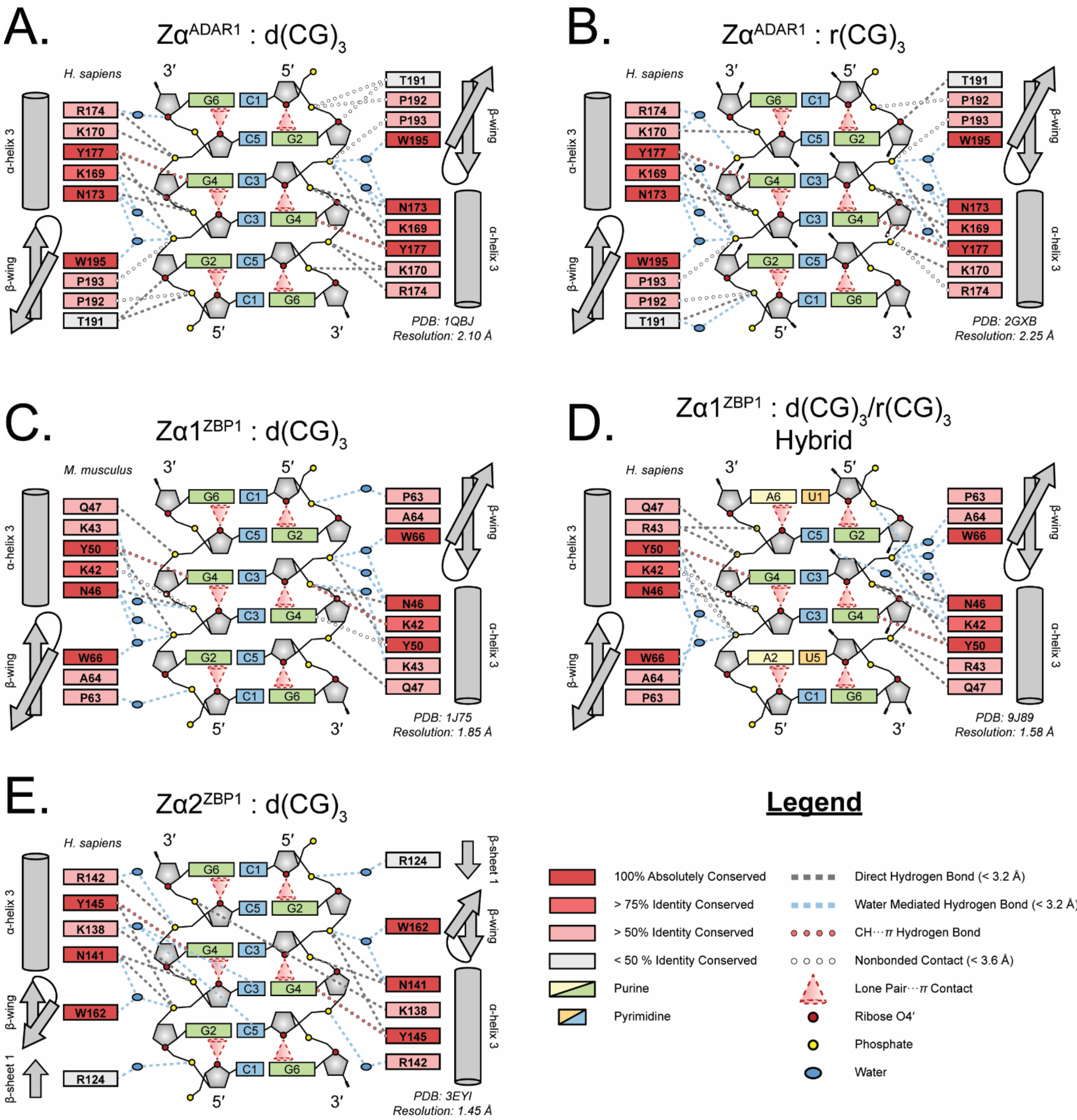

Supplementary Figure S1. Binding modes of mammalian Zα domains with DNA and RNA generated by NUCPLOT [1]. Moderately stringent optimal hydrogen bond distances (<3.2 Å), hydrogen bond geometries, and van der Waals contacts (<3.6 Å) were chosen because of the high resolution (<2.5 Å) structures available. **(A, B)** Binding mode of the Zα^{ADAR1} domain with self-complementary d(CpG)$_3$ and r(CpG)$_3$ oligos as seen in the crystal structures 1QBJ and 2GXB, respectively [2, 3]. **(C, D).** Binding mode of Zα1^{ZBP1} with a self-complementary d(CpG)$_3$ and a hybrid DNA:RNA oligo as seen in the crystal structures 1J75 and 9J89, respectively [4, 5]. It is important to note that our analysis of the crystal structures differ from those reported by the authors in [5], likely due to different restraints used in the analysis of bonds/contacts. **(E).** Binding mode of Zα2^{ZBP1} with a self-complementary d(CpG)$_3$ oligo as seen in the crystal structure 3EYI [6].

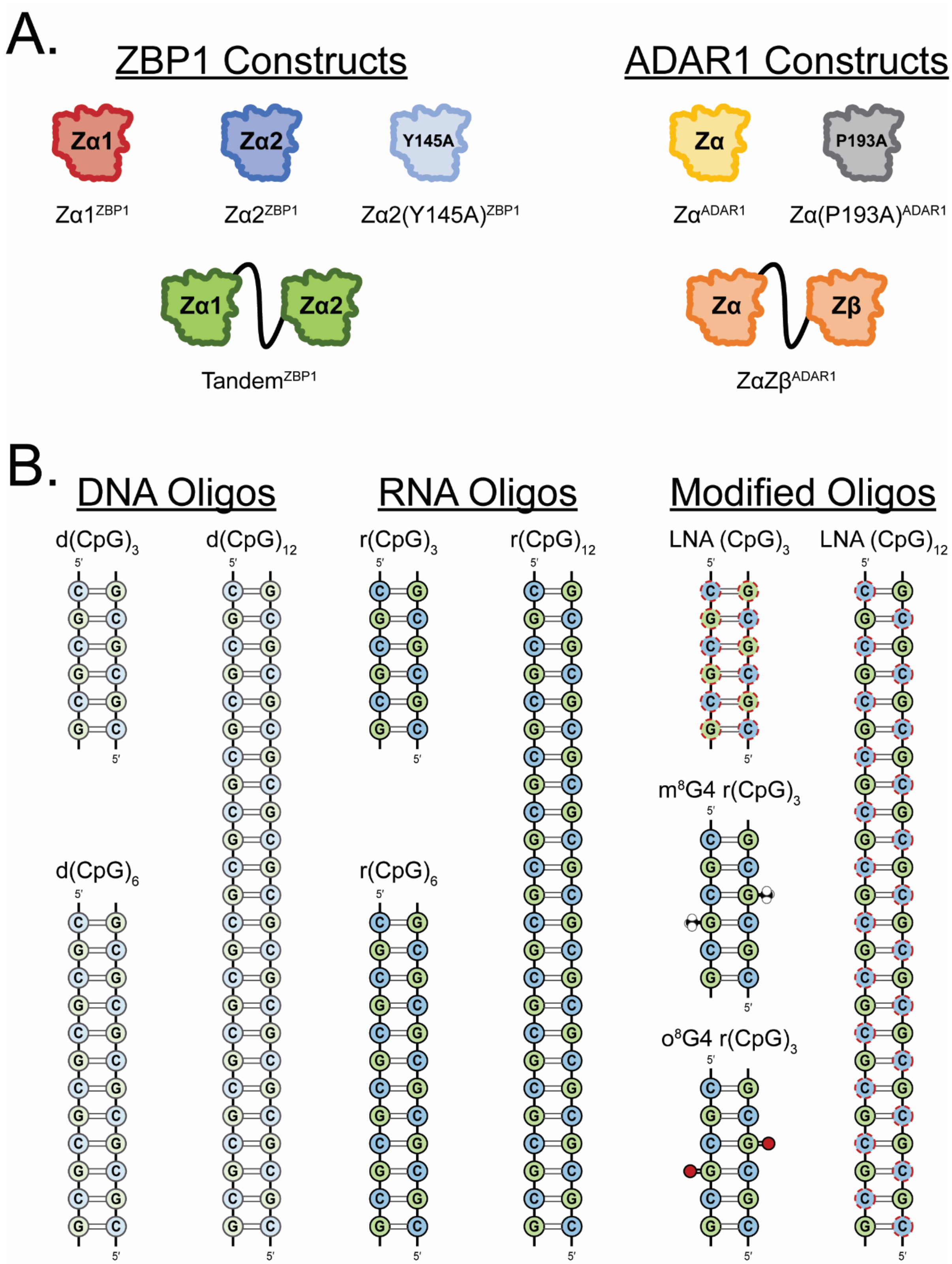

Supplementary Figure S2. Protein and oligo constructs. **(A)** ZBP1 and ADAR1 protein constructs and the names referenced throughout the manuscript. ZBP1 constructs include the isolated $Z\alpha1^{ZBP1}$ and $Z\alpha2^{ZBP1}$ domains, $Y145A^{ZBP1}$ in which the critical tyrosine residue is mutated and renders the domain nonfunctional, and $Tandem^{ZBP1}$ construct which contains both Zα1, Zα2, and the interconnected WT linker. ADAR1 constructs include the isolated $Z\alpha^{ADAR1}$ domain, $P193A^{ADAR1}$ in which one of the residues in the *cis-trans* proline-proline motif is mutated, and the tandem $Z\alpha Z\beta^{ADAR1}$ construct. Color-schemes for the proteins remain consistent throughout the manuscript. **(B)** Nucleic acid oligo constructs and the names referenced throughout the manuscript. All oligos are self-complimentary and were annealed immediately prior to measurements. Locked nucleic acid (LNA) sugar moieties are represented as red dashed circles. 8-methylguanine (m^8G) modifications are represented as black and white methyl space-filled models and 8-oxoguanine (o^8G) modifications are represented as red circles.

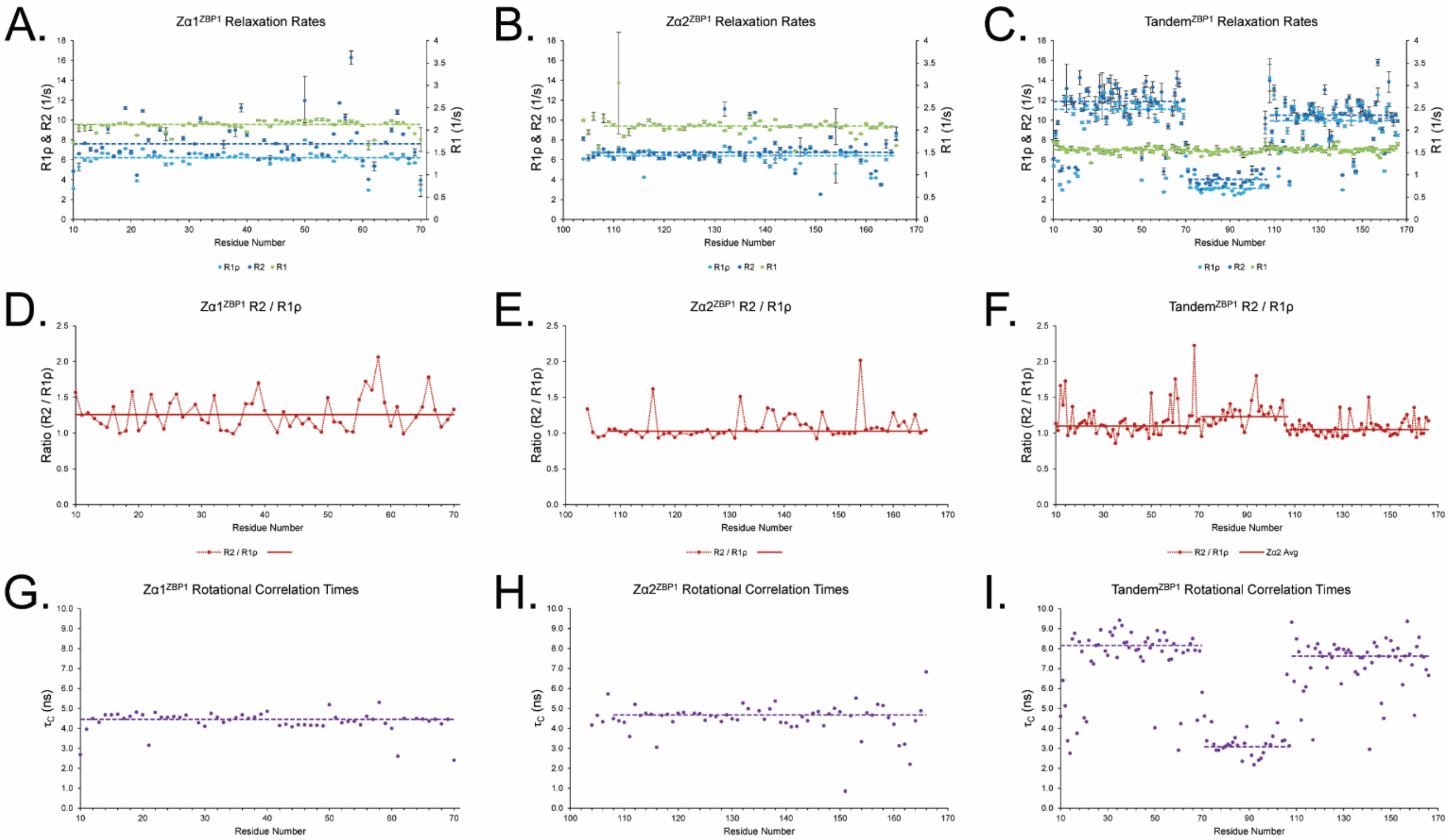

Supplementary Figure S3. Measured relaxation rates and calculated relaxation parameters of ZBP1 constructs. **(A-C)** Overlayed R2 (blue), R1ρ (cyan), and R1 (green) relaxation rates for each residue within the $Z\alpha1^{ZBP1}$, $Z\alpha2^{ZBP1}$, and $Tandem^{ZBP1}$ constructs, respectively. Average R2, R1ρ, and R1 rates reflect small, well-folded domains for the individual $Z\alpha1^{ZBP1}$ and $Z\alpha2^{ZBP1}$. The low R2 and R1ρ rates for the intrinsically disordered linker, as well as the larger rates for the ZBDs in the larger $Tandem^{ZBP1}$ construct reflect a tethered domain system with minimal interaction between ZBDs, and match previous reports [7]. **(D-F)** Ratio of R2 / R1ρ can be used to inform on regions experiencing increased R2 rates as a result of domain motions and/or exchange processes. $Z\alpha2^{ZBP1}$ has a region of increased $R2_{Ex}$ between residues 137-142, likely as a result of the dynamic folding/unfolding of a 3_{10} helix. Interestingly, the IDR linker of $Tandem^{ZBP1}$ also experiences increased R2 / R1ρ ratios, indicating contributions from exchange processes. **(G-I)** Calculated rotational correlation times (τ_C) of each ZBP1 construct. $Z\alpha1^{ZBP1}$ and $Z\alpha2^{ZBP1}$ τ_C averages (4.45 ± 0.07 ns and 4.66 ± 0.11 ns, respectively) reflect small, well-folded domains. Similarly, τ_C domain averages of $Tandem^{ZBP1}$ reflect a tethered domain system.

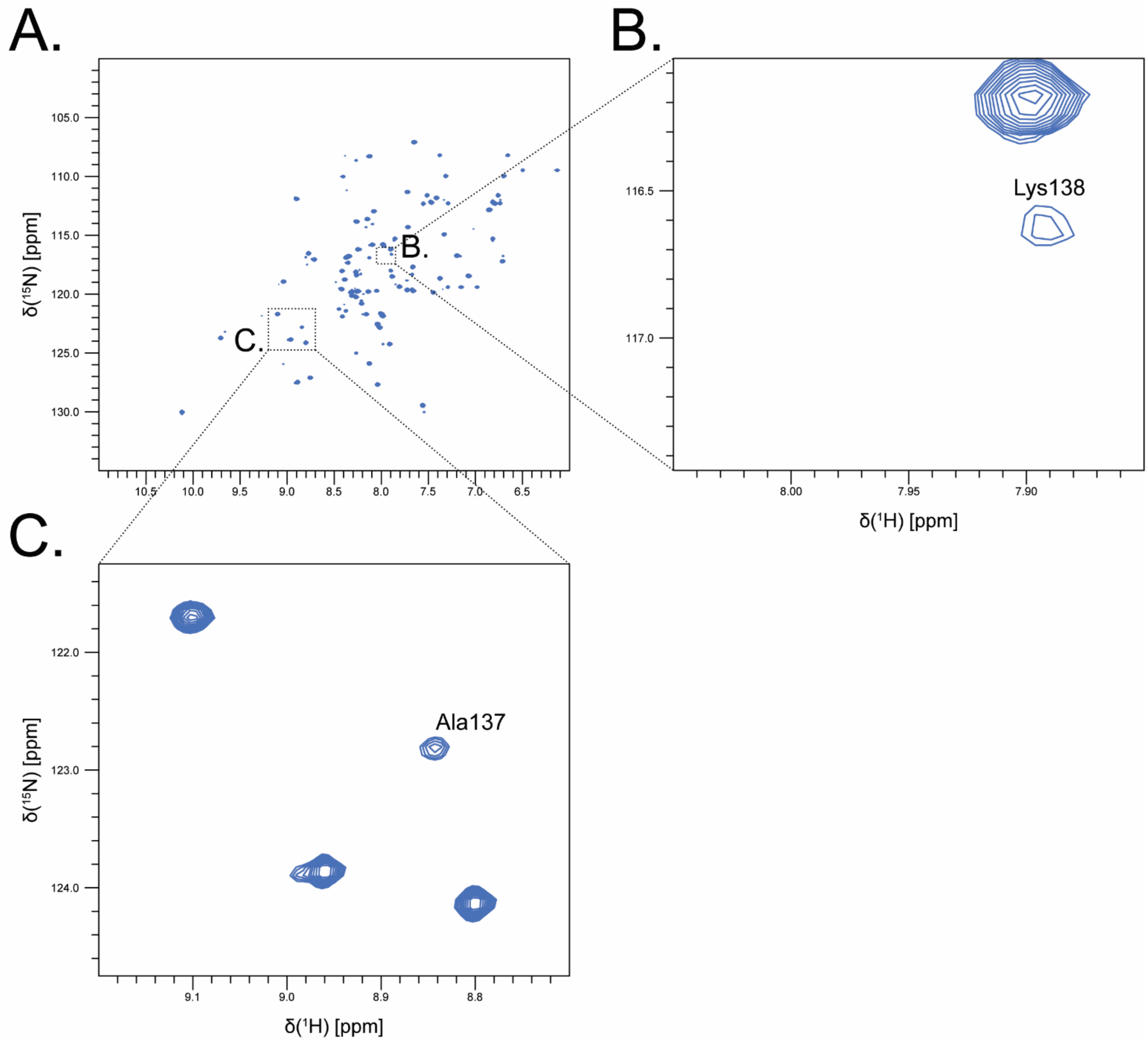

Supplementary Figure S4. ^{15}N-HSQC of Zα2^{ZBP1}. **(A)** Full Spectrum ^{15}N-HSQC of Zα2 shows many dispersed peaks, indicative of a well-folded protein. **(B-C)** Zoomed in regions surrounding Lys138 and Ala137, respectively. Both residues are heavily broadened compared to other peaks in the spectrum, indicating the presence of μs-ms exchange contributions.

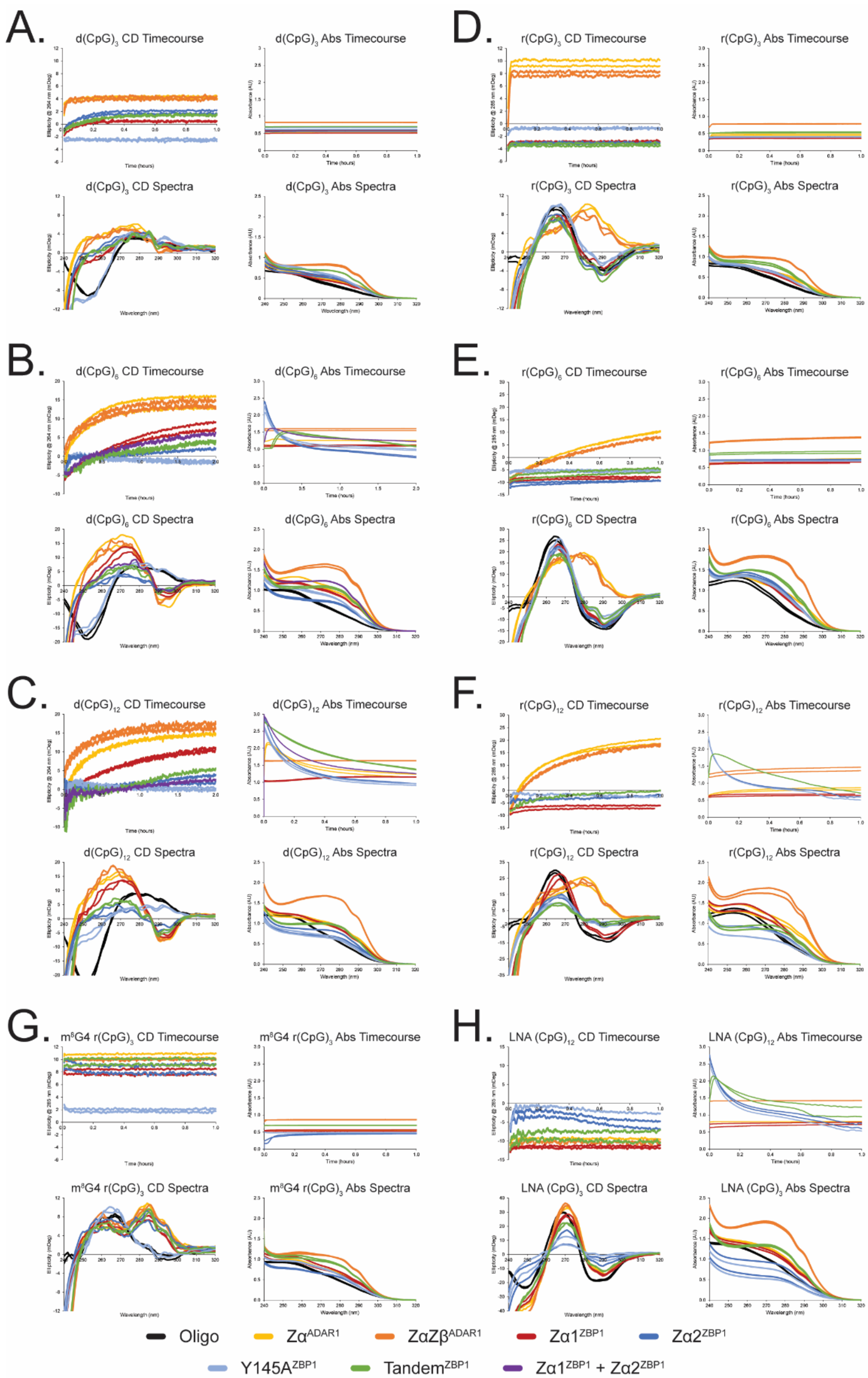

Supplementary Figure S5. Concurrent circular dichroism and absorbance measurements for each oligo tested in **(A-H)** under saturating protein concentrations. CD and absorbance timecourse measurements were taken simultaneously and reflect similar processes. CD and absorbance spectra were taken immediately after the timecourses. All plots represent the raw data collected except for the CD timecourses which instead depict the moving average (period of 20) of the data. **(A-C)** DNA oligos were measured at 15°C for at least one hour or until equilibrium was reached. **(D-F)** RNA oligos were measured at 42°C for at least 1 hour to roughly match the rate of the DNA oligos at 15°C according to previous findings [8]. **(G)** m^{8}G4 r(CpG) oligos were measured at 15°C in order to slow down the conversion rate. **(H)** LNA oligos were measured at 42°C.

S1. B- and A-to-Z Conversions are Comprised of Distinct Processes Occurring Simultaneously

Although we used pseudo first-order kinetics to model each ZBDs conversion of right-handed oligos for simplicity, we found that our CD data was best explained by fitting multi-exponential rate equations (Supplementary Table S1). The total number of potential rates varied from one to three depending on the protein construct and the length of the nucleic acid but could generally be classified into fast (≥ 20 hr^{-1}), intermediate (~10 hr^{-1}), or slow (< 5 hr^{-1}). The increased model complexity was justified using F-tests, as described in the methods section. The presence of multiple rates hints at different biological processes occurring simultaneously, although currently we can only hypothesize about what those processes are. In general, we assumed that the rate corresponding to the largest change in CD signal amplitude corresponds to the conversion rate as the right-to-left handed transition results in the largest difference in the CD spectra. A/B-to-Z conversion rates of the multi-exponential fits largely agreed with the single exponential rate of the pseudo-first order kinetics fits (Table 2, Supplementary Table S1). For the majority of oligos, the conversion rates fell within the slow processes, which agree with previous studies. Additionally, $Z\alpha1^{ZBP1}$, which lacks A-to-Z conversion activity, did not have a similar slow rate that would be indicative of flipping. For reasons similar to those encountered in the pseudo-first order kinetics analysis, many of the rates for $Z\alpha2^{ZBP1}$, $Y145A^{ZBP1}$, and $Tandem^{ZBP1}$ could not be fit due to the presence of biomolecular condensates interfering with measurements. The second and/or third rate constants accounted for much less of the overall amplitude of the data but greatly decreased the residuals of the fits.

The intermediate processes, when capable of being fit, accounted for the next largest contribution to the overall amplitude but were ~5x weaker on average. The rates for intermediate processes varied slightly across the measurable ZBD constructs; however, for each ZBD construct, there was a notable difference between DNA and RNA rates. On average, the intermediate rates for $Z\alpha^{ADAR1}$, $Z\alpha Z\beta^{ADAR1}$, and $Z\alpha1^{ZBP1}$ were 1.8x faster for DNA than for RNA (Supplementary Table S1), despite the RNA measurements being conducted at 42°C compared to the DNA measurements at 15°C. Z-RNA adoption has a much higher activation energy (3.2 $kcal \cdot mol^{-1} \cdot bp^{-1}$) compared to its DNA counterpart (2.0 $kcal \cdot mol^{-1} \cdot bp^{-1}$) [9], with much of this energy difference coming from the unfavorable adoption of the C2′-*endo* sugar pucker [10, 11]. Therefore, we think that this intermediate process is likely involved in the transient melting and/or rearrangement of the duplex into a Z-like state before full conversion to the Z-conformation. We also observed an extremely fast process that was dominant at the beginning of the measurements, but which accounted for the smallest percentage of the overall sign change in most cases outside of the short 6-mer oligos. When present, these rates were remarkably similar between all of the oligos for each ZBD, suggesting that the process governing it is inherent to the domain itself. This is corroborated by the fact that the rates are similar for DNA, RNA, and LNA regardless of each domain's conversion capabilities. Given the timescales (~20-150 hr^{-1}), these processes are unlikely to be involved in initial binding to the right-handed conformations but may still be involved in the nucleation of Z-forming regions.

Counterintuitively, the ADAR1 constructs, relative to the ZBP1 constructs, have extremely fast (~100 hr^{-1}) processes that also account for the majority of the signal when incubated with the short, 6-mer RNA and DNA oligos and a second, slower process (~10 hr^{-1}) that accounts for much less of the data. The ZBP1 constructs do not show the same trends with the 6-mer oligos; instead, they have a fast initial process with lower signal amplitudes, and a slower process that accounts for the majority of the signal, consistent with the longer oligos. It is currently unknown why the ADAR1 constructs display this characteristic, but it may be due to their ability to rapidly nucleate melted intermediates. The 6-mer DNA and RNA oligos have very low melting temperatures (T_M's) relative to the longer 12- and 24-mer oligos according to similar studies [8]. On the short oligos, $Z\alpha^{ADAR1}$ and $Z\alpha Z\beta^{ADAR1}$ may be able to fully melt the duplex and reform the strands into pseudo- or unstable Z-conformations. Under equilibrium conditions, these unstable complexes can slowly adopt more stable Z-conformations. Fitting multi-exponential rates, while much more complex, reveal consistent properties of each ZBD and their interactions with nucleic acids. However, the physical phenomenon underlying these rates is currently unknown, and future studies need to be conducted to more comprehensively understand the steps of Z-conversion.

Supplementary Table S1. Multi-Rate Exponential Fits of ZBD Constructs Extracted from Circular Dichroism Timecourses

	Oligo	# Fits	Amplitude 1	Amplitude 2	Amplitude 3	Rate 1 (hr^{-1})	Rate 2 (hr^{-1})	Rate 3 (hr^{-1})
$Z\alpha^{ADAR1}$	d(CG)3	Two-Rate	-2.40 ± 0.24	-0.72 ± 0.13		122.97 ± 12.36	11.18 ± 1.22	
	d(CG)6	Three-Rate	-12.87 ± 0.45	-2.51 ± 0.65	-2.51 ± 0.63	2.67 ± 0.05	10.17 ± 1.60	47.85 ± 9.69
	d(CG)12	Two-Rate	-9.77 ± 0.67	-7.22 ± 0.75		1.51 ± 0.10	7.18 ± 0.57	
	r(CG)3	One-Rate	-12.90 ± 0.22			96.97 ± 1.36		
	r(CG)6	Three-Rate	-25.17 ± 0.24	-3.82 ± 0.37	-0.98 ± 0.24	0.91 ± 0.02	4.97 ± 0.41	56.94 ± 12.03
	r(CG)12	Three-Rate	-12.15 ± 1.22	-12.24 ± 0.71	-6.83 ± 0.90	1.64 ± 0.07	5.47 ± 0.47	21.05 ± 1.51
	LNA (CG)12	One-Rate	-3.08 ± 0.28			104.26 ± 13.23		
$Z\alpha Z\beta^{ADAR1}$	d(CG)3	Two-Rate	-2.82 ± 0.35	-0.56 ± 0.16		109.15 ± 14.08	8.69 ± 1.94	
	d(CG)6	Two-Rate	-10.83 ± 0.23	-2.70 ± 0.43		2.17 ± 0.05	23.31 ± 3.96	
	d(CG)12	Three-Rate	-9.44 ± 0.56	-2.60 ± 0.65	-2.81 ± 1.18	2.25 ± 0.10	13.02 ± 3.89	163.57 ± 69.79
	r(CG)3	One-Rate	-11.77 ± 0.31			91.49 ± 1.99		
	r(CG)6	Two-Rate	-24.69 ± 0.20	-2.99 ± 0.36		0.85 ± 0.02	7.60 ± 0.93	
	r(CG)12	Two-Rate	-19.00 ± 0.47	-10.58 ± 0.51		1.82 ± 0.04	9.53 ± 0.42	
	LNA (CG)12	One-Rate	-3.03 ± 0.59			94.38 ± 25.59		
$Z\alpha1^{ZBP1}$	d(CG)3	One-Rate	-1.85 ± 0.08			11.58 ± 0.41		
	d(CG)6	Two-Rate	-14.99 ± 0.12	-0.97 ± 0.20		1.06 ± 0.02	8.94 ± 1.71	
	d(CG)12	Two-Rate	-13.15 ± 0.13	-1.68 ± 0.25		0.99 ± 0.02	15.69 ± 2.58	
	r(CG)3	One-Rate	-1.04 ± 0.13			58.96 ± 5.58		
	r(CG)6	Two-Rate	-1.26 ± 0.24	-0.99 ± 0.09		85.72 ± 15.78	6.08 ± 0.52	
	r(CG)12	Two-Rate	-1.00 ± 0.27	-1.54 ± 0.11		93.98 ± 25.33	6.99 ± 0.42	
	LNA (CG)12	One-Rate	-2.21 ± 0.34			96.79 ± 11.97		
$Z\alpha2^{ZBP1}$	d(CG)3	Two-Rate	-2.54 ± 0.14	-0.46 ± 0.22		8.59 ± 0.32	71.75 ± 34.92	
	d(CG)6	One-Rate	-4.66 ± 0.38			0.62 ± 0.07		
	d(CG)12							
	r(CG)3	One-Rate	-1.02 ± 0.15			65.84 ± 7.86		
	r(CG)6	Two-Rate	-2.38 ± 0.29	-1.74 ± 0.31		0.96 ± 0.13	132.62 ± 19.76	
	r(CG)12	Two-Rate	-0.99 ± 0.58	0.01 ± 0.04		3.12 ± 2.11	4.52 ± 1.84	
	LNA (CG)12	Two-Rate	-5.86 ± 0.54	21.50 ± 21.59		46.08 ± 3.95	0.21 ± 0.17	
$Y145A^{ZBP1}$	d(CG)3							
	d(CG)6	Two-Rate	-5.56 ± 0.34	2.28 ± 0.13		23.70 ± 1.48	1.11 ± 0.14	
	d(CG)12	Two-Rate	-9.16 ± 0.45	2.76 ± 0.26		23.62 ± 1.28	2.30 ± 0.21	
	r(CG)3	One-Rate	-1.21 ± 0.15			58.11 ± 6.11		
	r(CG)6	One-Rate	-0.84 ± 0.21			52.71 ± 10.49		
	r(CG)12							
	LNA (CG)12	Two-Rate	-8.16 ± 0.37	7.93 ± 5.32		44.61 ± 3.66	0.42 ± 0.36	
$Tandem^{ZBP1}$	d(CG)3	Two-Rate	-2.45 ± 0.09	-0.86 ± 0.35		5.82 ± 0.21	175.84 ± 68.18	
	d(CG)6	One-Rate	-8.81 ± 0.14			1.37 ± 0.04		
	d(CG)12	One-Rate	-12.62 ± 0.68			0.64 ± 0.05		
	r(CG)3	One-Rate	-0.94 ± 0.19			89.80 ± 14.93		
	r(CG)6	Two-Rate	-4.79 ± 0.14	-1.14 ± 0.20		0.94 ± 0.04	34.41 ± 5.47	
	r(CG)12	Two-Rate	-5.13 ± 0.38	-3.83 ± 0.75		1.79 ± 0.61	11.38 ± 2.59	
	LNA (CG)12	One-Rate	-2.55 ± 0.45			27.41 ± 4.05		

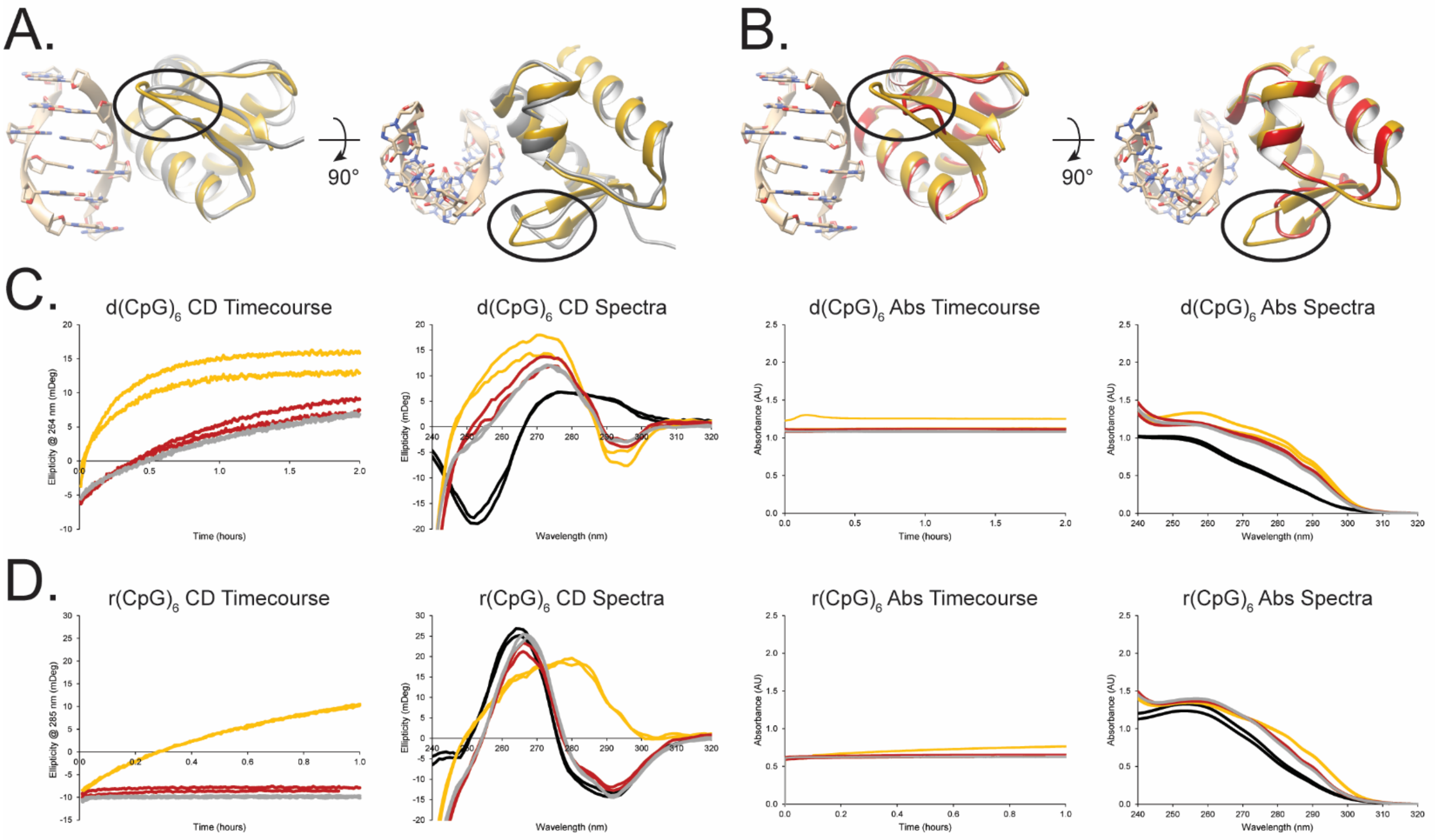

Supplementary Figure S6. *Cis-trans* proline-proline motif is critical for the β-wing fold and A-to-Z conversion activity. **(A)** Superposition of the crystal structure of ADAR1's Zα domain in complex with a d(CpG)$_3$ oligo (yellow; *PDB*: 1QBJ) with the NMR solution structure of P193A^{ADAR1} (grey; *PDB*: 8GBD) showcasing the misfolding of the β-wing (black circles) caused by the P193A point mutation. **(B)** Superposition of the same crystal structure (yellow; *PDB*: 1QBJ) with the crystal structure of the Zα1 domain of ZBP1 (red; *PDB*: 1J75) showcasing the different fold of the shortened β-wing (black circles). **(C-D)** CD and absorbance measurements of Zα^{ADAR1} (yellow), Zα1^{ZBP1} (red), and P193A^{ADAR1} (grey) with d(CpG)$_6$ and r(CpG)$_6$ oligos (C and D, respectively). Experimental conditions were the same as in Supplementary Figure S5 and black lines represent the spectra of the free oligos.

S2. Zα2 Drives Biomolecular Condensation that is Independent of Z-Conversion Activity

Microscopic morphological analysis revealed that most Zα2-containing constructs formed highly circular condensates when incubated with 12-mer or 24-mer DNA and RNA oligos at 15°C (Supplementary Figure S7). Interestingly, $Z\alpha2^{ZBP1}$, as opposed to $Y145A^{ZBP1}$, did not form visible particles when incubated with $r(CpG)_6$ at 15°C (Supplementary Figure S7A), instead requiring a lower temperature of ~4°C for the formation of particles to be apparent (data not shown). Additionally, $Tandem^{ZBP1}$ did not form particles when incubated with either the 12-mer or 24-mer RNA oligos, suggesting that the $Tandem^{ZBP1}$ construct's ability to form particles may be more sensitive to differences in starting conditions (stock concentrations, pre-mixing temperatures, surface wetting, etc.). Furthermore, optical tweezer manipulation revealed that $Z\alpha2^{ZBP1}$ – $d(CpG)_{12}$ and $Z\alpha2^{ZBP1}$ – $r(CpG)_{12}$ particles were highly spherical when suspended in solution and readily fused with one another (Supplementary Videos S1 and S2). The resulting fusions quickly returned to being highly spherical, with the measured radii closely matching the predicted radii, as expected from the addition of two spherical volumes (Supplementary Figure S7B). These results, in addition to extracted fusion times (τ_F; Supplementary Figure S7C), suggest that the particles formed by the incubation of Zα2-containing ZBP1 constructs with the longer DNA and RNA oligos are liquid-like in nature [12]. It is interesting to note that fusion dynamics were different between DNA and RNA oligos and seemed to change over the course of several hours. However, further characterizations are necessary to determine the exact mechanisms driving the liquid-like biomolecular condensation (LLBC).

Although all Zα2-containing ZBD constructs formed LLBCs with the longer oligos under CD experimental conditions, each construct demonstrated different nucleation and settling rates apparent in the absorbance timecourse measurements (Supplementary Figure S5B,C,F,H). Absorbance measurements were taken simultaneously with the CD timecourse measurements and provided additional information on the interactions between the ZBD constructs and the oligonucleotides. The nucleation of liquid-like droplets resulted in an initial increase in the absorbance measured as the light was scattered by the large droplets rather than being absorbed by the macromolecules in solution. The droplets, being denser than water, slowly settled out of solution over the course of the experiment, resulting in a slow decrease in light scattering and thus an apparent decrease in absorbance. Similar settling of the droplets out of solution were observed in the optical tweezer experiments. When $Z\alpha2^{ZBP1}$ and $Y145A^{ZBP1}$ were incubated with the $d(CpG)_6$ oligo at 15°C, LLBCs immediately formed and began to rapidly settle out of solution (Supplementary Figure S5B). Interestingly, when the $Tandem^{ZBP1}$ construct was mixed with the same oligo, there was an initial lag period of ~6 min before particles formed, suggesting that the presence of Zα1 negatively affected the initiation of LLBC. Furthermore, nucleation of LLBC and the settling of the $Tandem^{ZBP1}$ droplets occurred much more slowly than for the isolated Zα2 constructs, indicating that the smaller domains form larger or more dense condensates than the tandem domains. Indeed, microscopic analysis showed that incubation of $Z\alpha2^{ZBP1}$ or $Y145A^{ZBP1}$ with the longer oligos resulted in increased surface wetting when compared to $Tandem^{ZBP1}$ (Supplementary Figure S7A), suggesting that the isolated domains settled to the bottom of the plate faster than the tandem construct. To confirm that Zα1 had a negative effect on Zα2-driven LLBC, we also incubated equimolar amounts of $Z\alpha1^{ZBP1}$ and $Z\alpha2^{ZBP1}$ with the $d(CpG)_6$ oligo. The presence of $Z\alpha1^{ZBP1}$ in *trans* slowed down the nucleation and settling of the droplets, however not to the same extent as $Tandem^{ZBP1}$, confirming Zα1's negative effect on droplet formation that is enhanced when ZBP1's ZBDs are expressed in tandem.

Doubling the length of the DNA oligo to 24 bps resulted in a drastic increase in the nucleation of droplets (Supplementary Figure S5C). With the $d(CpG)_{12}$ oligo, $Z\alpha2^{ZBP1}$, $Y145A^{ZBP1}$, and $Tandem^{ZBP1}$ had all nucleated LLBC immediately upon mixing and before the measurement could be started, suggesting that droplet formation was partially dependent on oligo length. Despite similar fast nucleation times, the Zα2-containing constructs displayed similar trends in their settling rates, with $Z\alpha2^{ZBP1}$ and $Y145A^{ZBP1}$ being nearly identical and having the fastest setting rates, followed by equimolar combinations of $Z\alpha1^{ZBP1}$ and $Z\alpha2^{ZBP1}$, and finally $Tandem^{ZBP1}$. Interestingly, $Z\alpha^{ADAR1}$ also displayed an increase in absorbance that could be indicative of LLBC; however, no cloudiness or partitioning was observed at the end of the measurements, indicating that the increase in absorbance was due to a separate phenomenon or that $Z\alpha^{ADAR1}$ was able to resolve its droplets by the end of the measurement period.

Interestingly, droplet formation was not observed when any Zα2-containing protein construct was incubated with the $r(CpG)_6$ oligo at 42°C as opposed to the $d(CpG)_6$ oligo at 15°C. Additionally, lowering the temperature of the $Z\alpha2^{ZBP1}$ $r(CpG)_6$ incubation to ~4°C resulted in the appearance of droplets, indicating that Zα2-driven biomolecular condensates may display Upper Critical Solution Temperature (UCST), as opposed to Lower Critical Solution Temperature (LCST), behavior. UCST phase transitions are enthalpically driven [13], in which specific interactions between charged, polar, and aromatic residues play a crucial role [14]. Indeed, while DLS measurements of the individual components (Supplementary Figure S8A) showed that they were predominantly monomeric in solution, incubations of the Zα2-containing constructs with the 12-mer and 24-mer DNA and RNA oligos immediately before (15°C) and after (42°C) thermal heat ramps showed increased turbidity and light scattering indicative of large particles at low temperatures that largely disappeared as the temperature was increased (Supplementary Figure S8B-D). Additionally, the disappearance and reappearance of the large droplets was reversible with increasing and decreasing the temperature of the samples, respectively (Supplementary Figure S8E-G), which is indicative of both the formation of biomolecular condensates and of UCST behavior. Doubling the length of the $r(CpG)_6$ oligo to 24 bps results in a return of particle formation confirming that LLBC is length dependent and indicates that the UCST can be increased with length as well. The condensation of the $r(CpG)_{12}$ oligo showed similar characteristics to the DNA 12- and 24-mers in which $Z\alpha2^{ZBP1}$ and $Y145A^{ZBP1}$ showed nearly identical nucleation and settling rates and where $Tandem^{ZBP1}$ had much slower nucleation and settling rates. The same trends in absorbance measurements were observed with the LNA $(CpG)_{12}$, once again confirming that flipping ability is not necessary for the initiation of LLBC.

It is also important to note that all ZBDs displayed a minor gradual increase in absorbance over the course of the CD measurements (Supplementary Figure S5). These increases could be due to differences in base stacking interactions between the A/B-

and Z-conformations that affect their respective electronic excitations, leading to differences in the UV-Vis absorbance profiles. This could be the case for the RNA oligos, as there is a moderate increase in the absorbance of the Z-RNA spectrum at 285 nm when compared to the A-RNA absorbance spectrum; however, for the DNA oligos, there is little to no change in the absorbance spectra at 264 nm [15]. Additionally, Zα1^{ZBP1}, which neither forms noticeable particles nor flips RNA to the Z-conformation, similarly shows a moderate increase in absorbance over the course of the experiment. Alternatively, the increase in absorbance could be due to the generation and maintenance of a melted intermediate species. Both DNA and RNA experience hyperchromic effects when transitioning from ordered, native structures to disordered, denatured structures. Extinction coefficients increase across the visible spectrum for denatured oligos, however, maximal increases for GC-rich oligos occur around 280 nm for both DNA and RNA [16, 17]. As previously discussed, right-to-left-handed conversion of nucleic acids proceeds through melted intermediates [8], and thus, the increases in absorbance that we see for all oligos could be in part caused by the promotion or adoption of melted intermediates.

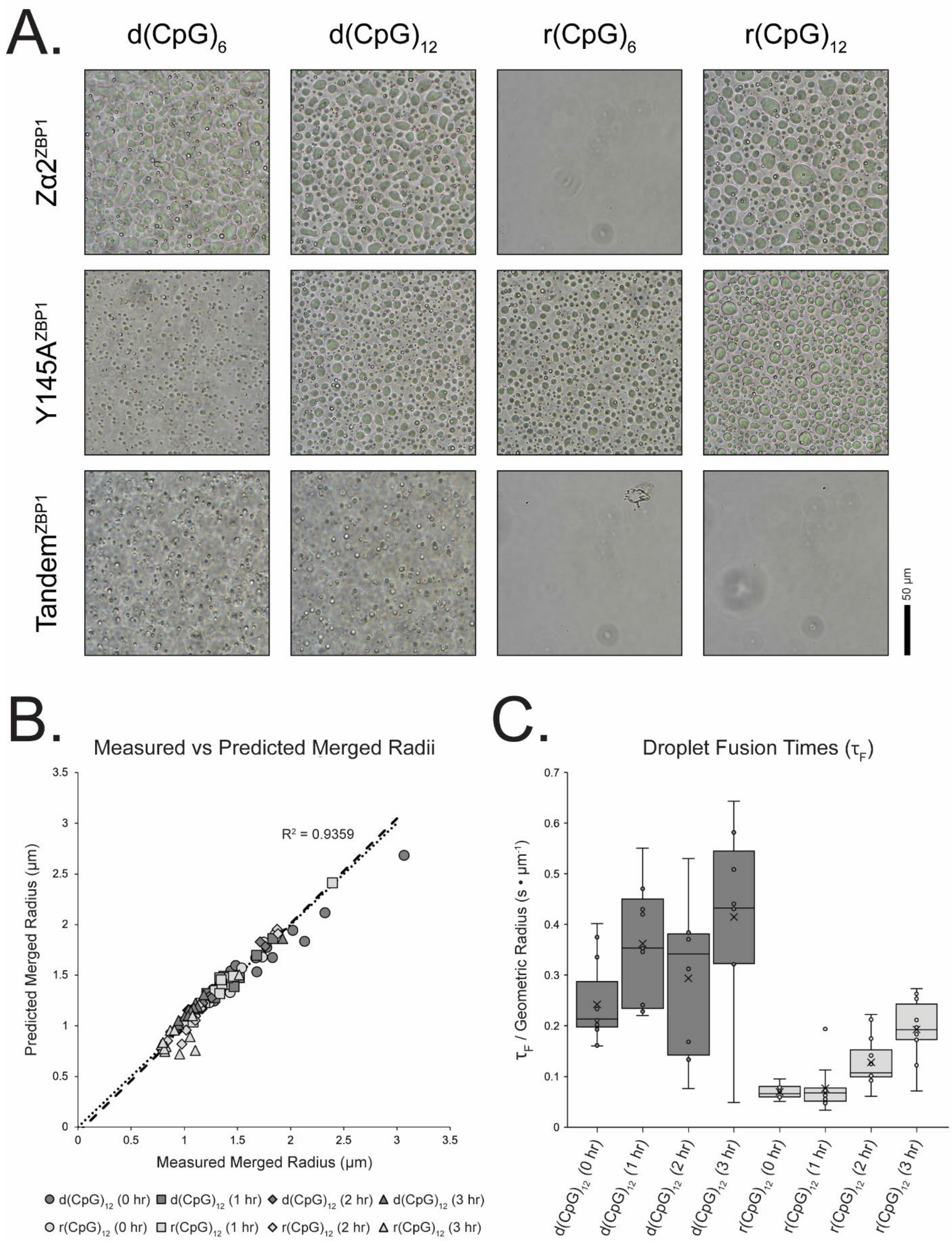

Supplementary Figure S7. Morphological and biophysical analysis of Zα2-driven liquid-like biomolecular condensation. **(A)** 40X magnification images of different Zα2-containing ZBP1 constructs when incubated with $d(CpG)_6$, $d(CpG)_{12}$, $r(CpG)_6$, and $r(CpG)_{12}$ oligos at 15°C. **(B)** Predicted versus measured radii of $Z\alpha2^{ZBP1}$ – $d(CpG)_{12}$ and $Z\alpha2^{ZBP1}$ – $r(CpG)_{12}$ droplets after merger events using optical tweezer manipulation. Ideal (dotted line) and actual (dashed line) correlations between predicted and measured radii are plotted. **(C)** Droplet Fusion Times (τ_F) corrected for geometric radius for $Z\alpha2^{ZBP1}$ – $d(CpG)_{12}$ and $Z\alpha2^{ZBP1}$ – $r(CpG)_{12}$ droplets over the course of 3 hours.

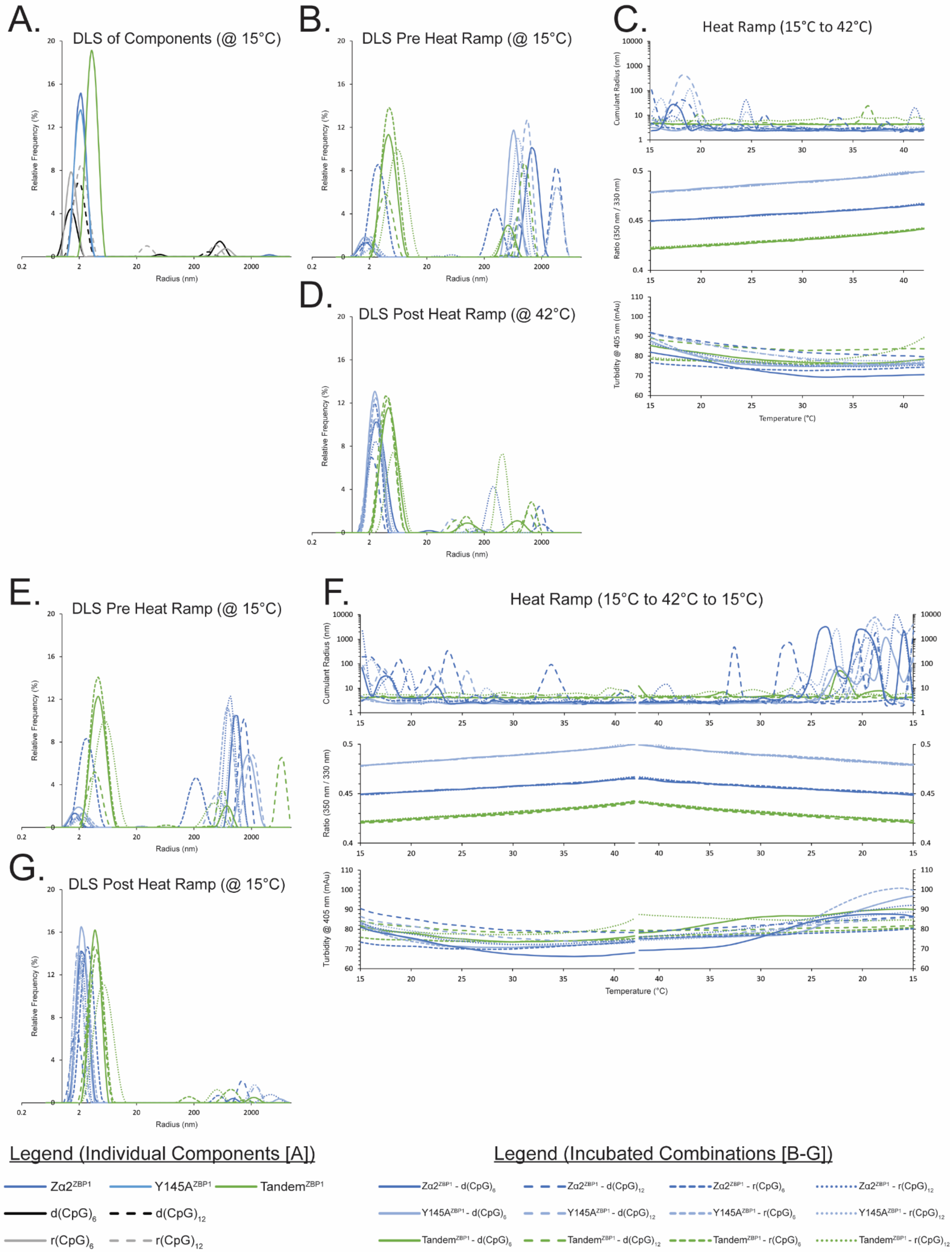

A.
DLS of Components (@ 15°C)
B.
DLS Pre Heat Ramp (@ 15°C)
C.
Heat Ramp (15°C to 42°C)
D.
DLS Post Heat Ramp (@ 42°C)
E.
DLS Pre Heat Ramp (@ 15°C)
F.
Heat Ramp (15°C to 42°C to 15°C)
G.
DLS Post Heat Ramp (@ 15°C)
Relative Frequency (%)
Radius (nm)
Cumulant Radius (nm)
Ratio (350 nm / 330 nm)
Turbidity @ 405 nm (mAu)
Temperature (°C)
Legend (Individual Components [A])
Zα2ZBP1
Y145AZBP1
TandemZBP1
d(CpG)6
d(CpG)12
r(CpG)6
r(CpG)12
Legend (Incubated Combinations [B-G])
Zα2ZBP1 - d(CpG)6
Zα2ZBP1 - d(CpG)12
Zα2ZBP1 - r(CpG)6
Zα2ZBP1 - r(CpG)12
Y145AZBP1 - d(CpG)6
Y145AZBP1 - d(CpG)12
Y145AZBP1 - r(CpG)6
Y145AZBP1 - r(CpG)12
TandemZBP1 - d(CpG)6
TandemZBP1 - d(CpG)12
TandemZBP1 - r(CpG)6
TandemZBP1 - r(CpG)12

Supplementary Figure S8. Dynamic Light Scattering (DLS) measurements of different Zα2-containing ZBP1 constructs with 12-mer and 24-mer DNA and RNA oligos. **(A)** Control DLS of isolated components (see corresponding legend) before mixing shows the lack of protein multimerization at the concentrations used. Oligo multimerization was present, likely as a result of laddering between the self-complimentary single-stranded oligonucleotides. **(B-D)** DLS of incubated combinations (see corresponding legend) immediately after mixing at 15°C **(B)**, followed by concurrent measurement of DLS (cumulant radius; top), protein unfolding (Ratio [350 nm / 330 nm]; middle) and turbidity (405 nm; bottom) as temperature was increased from 15°C to 42°C **(C)**, and DLS of incubated combinations at 42°C **(D)**. **(E-G)** Same as **B-D** with the addition of a temperature ramp down (42°C to 15°C) immediately after the temperature ramp up. Settling and adsorption of droplets on the capillary surface and/or combinations of droplets resulting in particles > 1 μm resulted in the disappearance of measured particles as they fell outside of DLS detection limits.

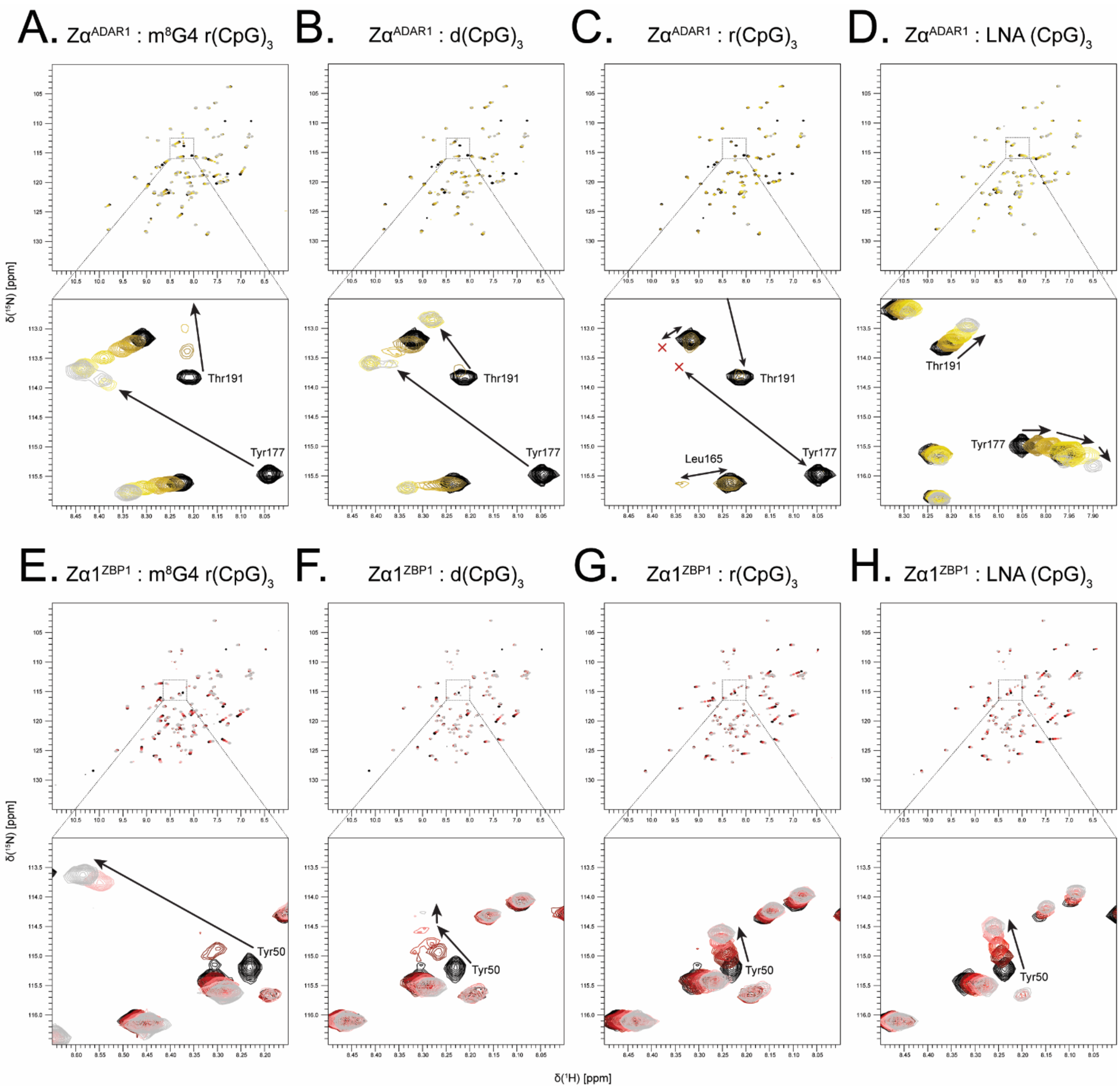

Supplementary Figure S9. ^{15}N-HSQC titration series of the representative ZBDs of ADAR1 and ZBP1 with different 6-mer DNA/RNA oligos. **(A-D)** Titration series of Zα^{ADAR1} with the Z-form m^{8}G4 r(CpG)$_3$, B-form d(CpG)$_3$, A-form r(CpG)$_3$, and A-Like LNA (CpG)$_3$ oligos, respectively. Titration points are color coded from black (Apo), through shades of yellow (1:0.25, 1:0.50, 1:1, 1:2), to grey (1:4). Inset boxes highlight residues Tyr177 and Thr191 which show significant peak shifts and are critical for flipping abilities. Tyr177 and Thr191 in the r(CpG)$_3$ series (red crosses) shift to similar positions as seen with the m^{8}G4 r(CpG)$_3$ and d(CpG)$_3$ residues but are heavily broadened. **(E-H)** Titration series of Zα1^{ZBP1} with the same oligos as shown in (A). Titration points are color coded from black (Apo), through shades of red (1:0.25, 1:0.50, 1:1, 1:2), to grey (1:4). Inset boxes highlight the CSPs of the critical Tyr50 residue.

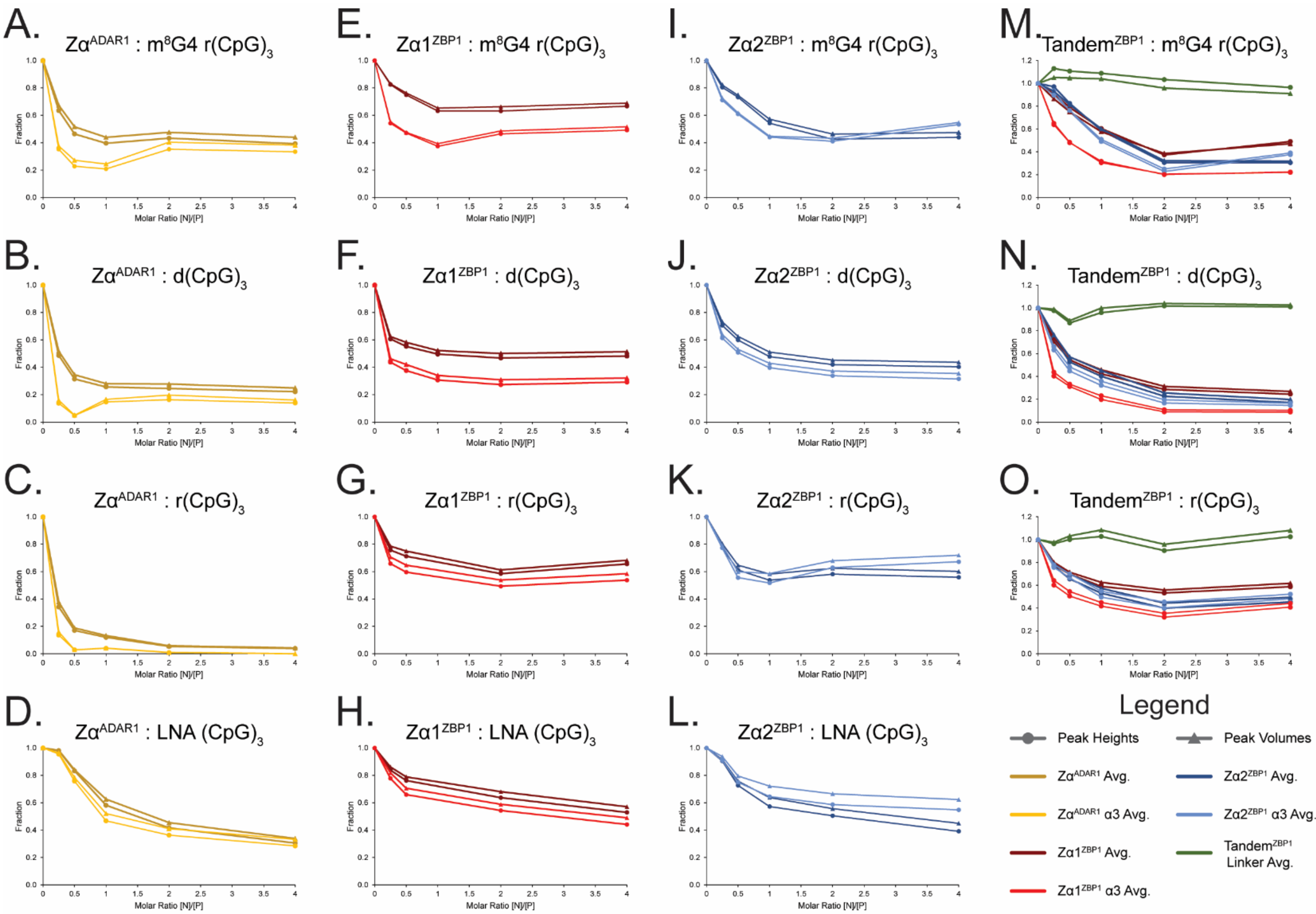

Supplementary Figure S10. Average peak heights and volumes for amide signals in each ZBD – oligo ^{15}N-HSQC titration series represented by circles and triangles, respectively. **(A-D)** Average peak heights and volumes of each Zα^{ADAR1} titration series across the entire domain (gold) and across just the α3-helix (yellow). **(E-H)** Average peak heights and volumes of each Zα1^{ZBP1} titration series across the entire domain (maroon) and across just the α3-helix (red). **(I-L)** Average peak heights and volumes of each Zα2^{ZBP1} titration series across the entire domain (navy) and across just the α3-helix (blue). **(M-O)** Average peak heights and volumes of each Tandem^{ZBP1} titration series across the entire Zα1 domain (maroon) or across Zα1's α3-helix (red), across the entire IDR linker (green), and across the entire Zα2 domain (navy) or across Zα2's α3-helix (blue).

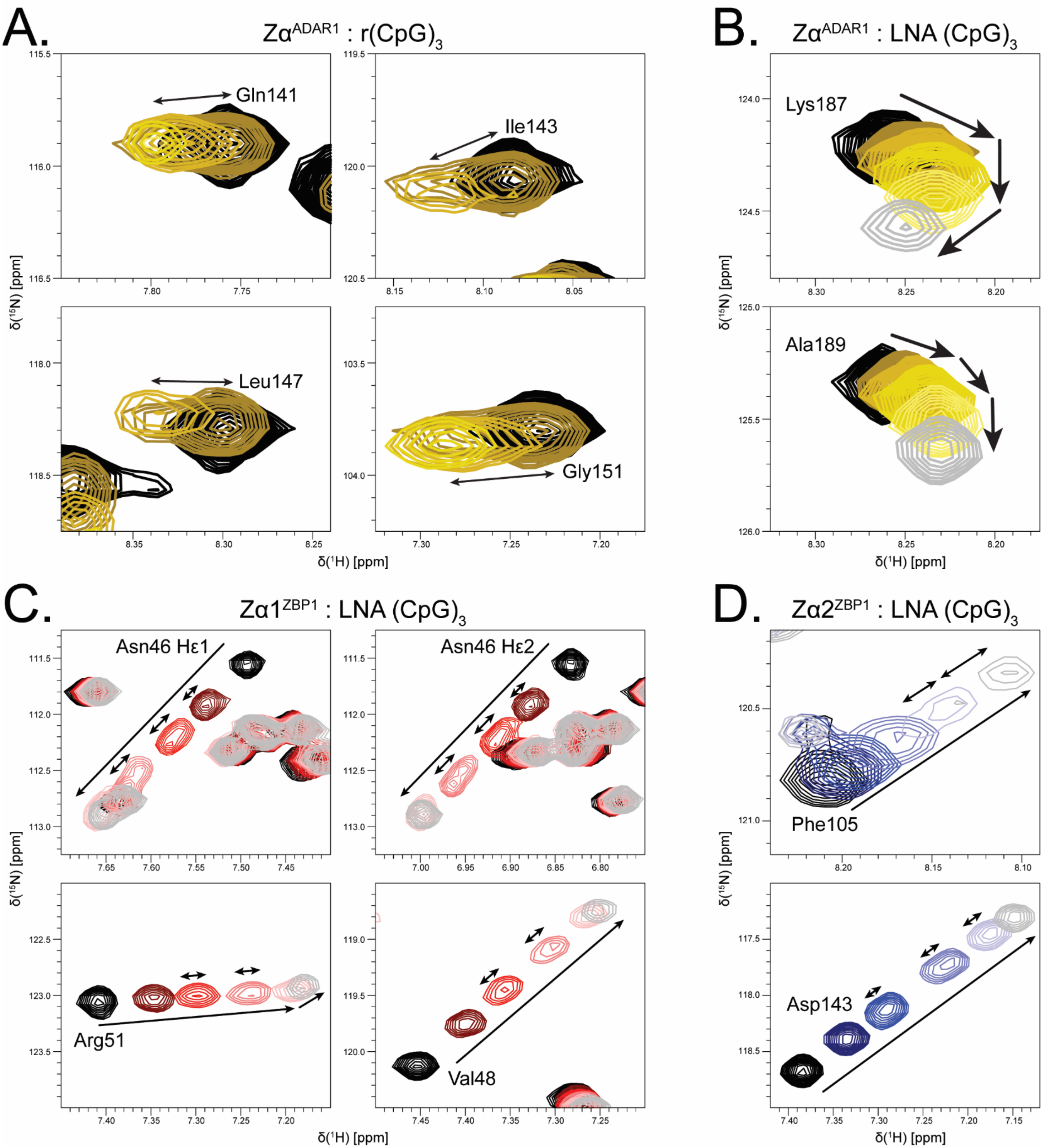

Supplementary Figure S11. Atypical shift behaviors identified in the ADAR1 and ZBP1 oligo titrations. **(A)** Characteristic examples of slow exchange in the $Z\alpha^{ADAR1}$ – r(CpG)$_3$ ^{15}N-HSQC titration series. The amide signal of free protein (black) slowly disappears, and the amide signal of bound protein (shades of yellow and grey) slowly appears as oligo concentrations are increased. **(B)** The $Z\alpha^{ADAR1}$ – LNA ^{15}N-HSQC titration series show nonlinear, multimodal binding modes consistent with multiple oligos binding the same ZBD [18]. **(C, D)** Amide signals in the interaction interface of $Z\alpha1^{ZBP1}$ (red, **C**) and $Z\alpha2^{ZBP1}$ (blue, **D**) show both fast and slow exchange processes at intermediate titration points in the LNA (CpG)$_3$ titration series.

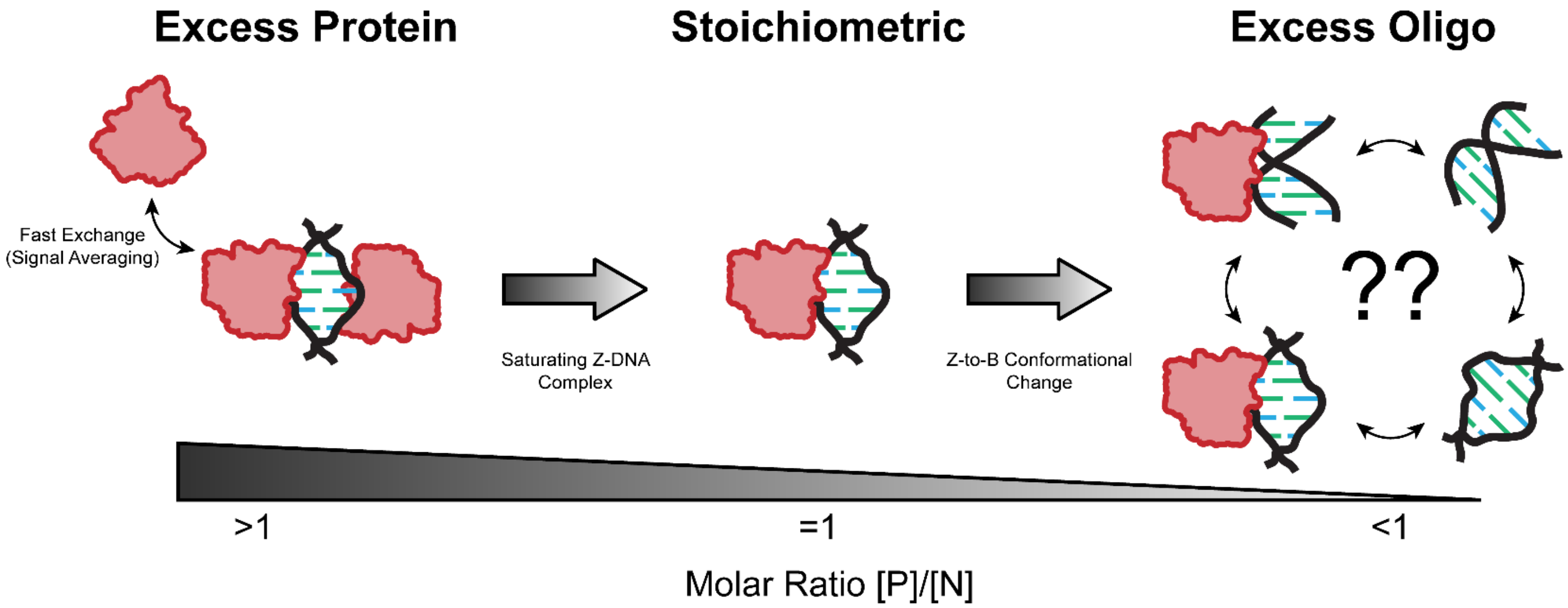

Supplementary Figure S12. Graphical model explaining bimodal binding shifts seen in ZBP1 – d(CpG)$_3$ oligo titrations. Under protein saturating conditions, ZBP1 ZBDs are able to fully saturate the B-DNA oligos and convert them to the Z-conformation. Fast/intermediate exchange between free and bound ZBDs reduce the overall magnitude of shifts at early titration points. As nucleic acid concentrations increase up to equimolar stoichiometries, ZBD – Z-DNA complexes are saturated, and first binding mode reaches maximal shifts. Because ZBP1's ZBDs form unstable complexes with short 6-mer DNAs, as oligo concentrations are further increased, ZBDs begin to be titrated off and lose the ability to stabilize the Z-conformation, resulting in a switch to the second, right-handed binding mode. It is currently unclear whether the oligos revert on the ZBD or if dissociation from the ZBD results in a reversion to the B-form.

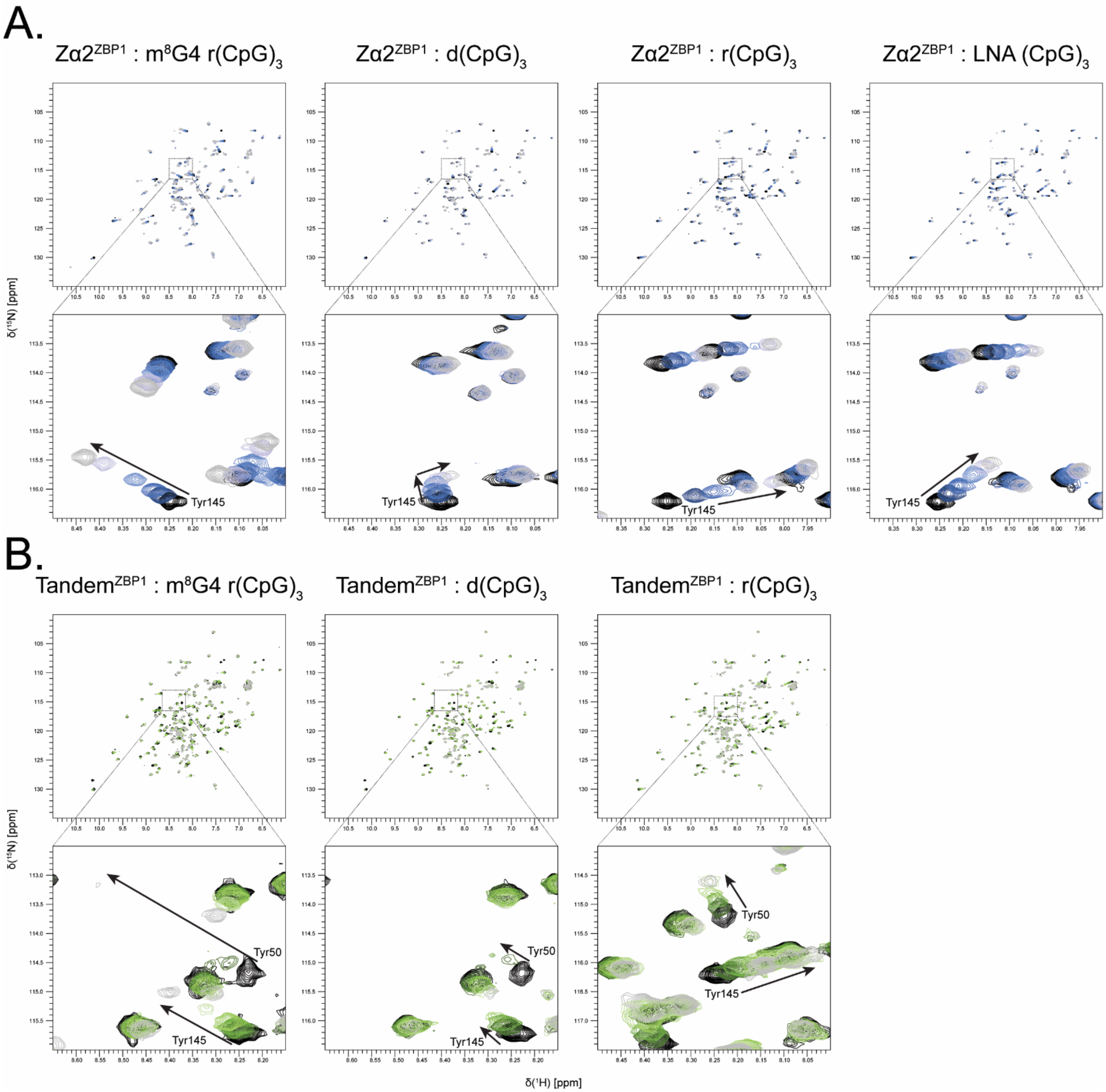

Supplementary Figure S13. ^{15}N-HSQC titration series of $Z\alpha2^{ZBP1}$ and Tandem^{ZBP1} with different 6-mer DNA/RNA oligos measured at 35°C. **(A)** Titration series of $Z\alpha2^{ZBP1}$ with the Z-form m^{8}G4 r(CpG)$_3$, B-form d(CpG)$_3$, A-form r(CpG)$_3$, and A-Like LNA (CpG)$_3$. Titration points are color coded from black (Apo), through shades of blue (1:0.25, 1:0.50, 1:1, 1:2), to grey (1:4). Inset boxes highlight residues Tyr145 which show significant peak shifts and are critical for flipping abilities. $Z\alpha2^{ZBP1}$ – d(CpG)$_3$ titration series has bimodal binding modes similar to $Z\alpha1^{ZBP1}$. **(B)** Titration series of Tandem^{ZBP1} with the same oligos seen in (A) with the exception of the LNA (CpG)$_3$. Titration points are color coded from black (Apo), through shades of green (1:0.25, 1:0.50, 1:1, 1:2), to grey (1:4). Inset boxes highlight the CSPs of the critical Tyr50 and Tyr145 residues of Zα1 and Zα2, respectively. Peaks for Zα1 and Zα2 shift to similar positions as seen with the $Z\alpha1^{ZBP1}$ and $Z\alpha2^{ZBP1}$ titration series but are heavily broadened due to increased complex size.

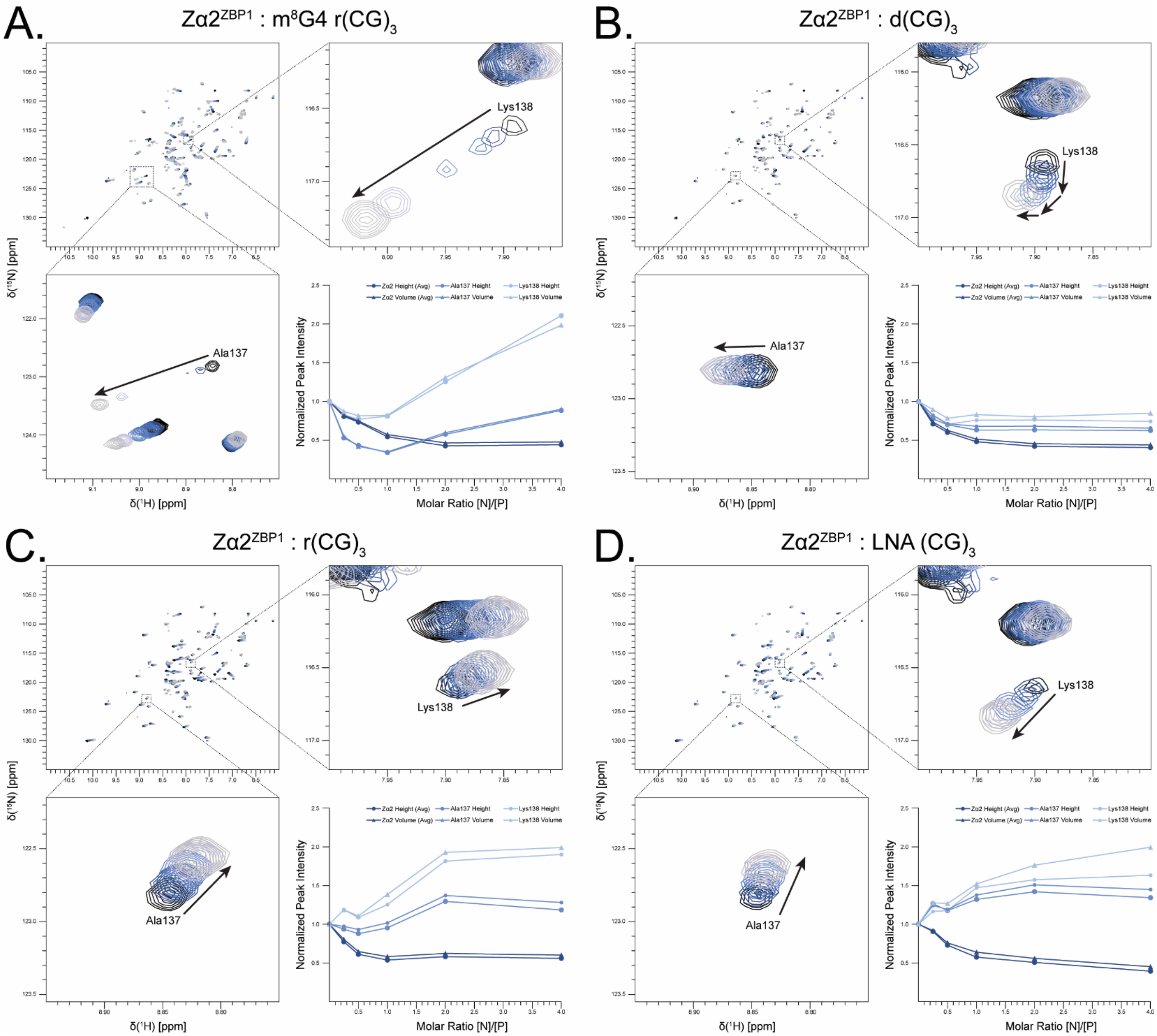

Supplementary Figure S14. Intermediate exchange seen in residues within the 3_{10}-helix at the N-terminus of Zα2[ZBP1]'s recognition helix is quenched upon addition of increasing concentrations of m[8]G4 r(CpG)$_3$ **(A)**, r(CpG)$_3$ **(C)**, and LNA (CpG)$_3$ **(D)** oligos. Titration of d(CpG)$_3$ **(B)** oligo into Zα2[ZBP1] does not quench intermediate exchange, but displays unique, multimodal binding modes for Lys138.

S3. 1D Imino Proton Spectra Confirm the Adoption of A-, B-, or Z-Conformations Throughout ZBD Titrations

Supplementary Figure S15A-D shows changes in the 1D imino proton spectra of the m^8G4 r(CpG)$_3$, d(CpG)$_3$, r(CpG)$_3$, and LNA (CpG)$_3$ oligos upon titration of the different ADAR1 and ZBP1 ZBD constructs. The imino resonances were assigned using previous assignments from studies of the d(CpG)$_3$ and r(CpG)$_3$ oligos [19] and LNA (CpG)$_3$ oligos [18]. Assignments of the free m^8G4 r(CpG)$_3$ imino resonances were inferred from their similarity to 1D imino spectra of the d(CpG)$_3$ and r(CpG)$_3$ oligos under saturating $Z\alpha^{ADAR1}$ concentrations, assuming that the m^8G4 r(CpG)$_3$ oligo is adopting a substantial Z-form population under our experimental conditions. Similar to previous reports [8], the m^8G4 r(CpG)$_3$ is transiently sampling both the A- and Z-conformations or a melted intermediate at 35°C in the absence of protein as the imino resonances for G2 is nonexistent and G4 is heavily broadened, indicating accessibility to and exchange with water which decreases signal intensity (Supplementary Figure S15A). The addition of any of the ZBD constructs to m^8G4 r(CpG)$_3$ resulted in a relative increase in the G4 imino signal, and the potential appearance of G2 at early titration points when protein saturating conditions allowed for the full stabilization of the duplexed RNA and the quenching of exchange with water. m^8G4 r(CpG)$_3$ imino resonances for all ZBD titrations additionally displayed fast exchange regimes throughout the titration, which largely match the exchange regimes seen for the 2D ^{15}N-HSQC titration series, confirming the relatively fast interconversion of the m^8G4 modification between the A- and Z-conformations. Interestingly, increasing relative concentrations of $Z\alpha^{ADAR1}$ did not cause significant CSPs in the G4 resonance, possibly indicating the adoption of similar Z-conformations in the presence and absence of protein. However, relative increases in $Z\alpha1^{ZBP1}$ or $Z\alpha2^{ZBP1}$ concentrations did result in significantly different downfield and upfield imino resonances, respectively, potentially suggesting different free- and protein-bound Z-RNA conformations. Addition of Tandem^{ZBP1} to the m^8G4 r(CpG)$_3$ oligo resulted in minor downfield shifts perturbations to the G4 imino resonance. The lower overall shifts are likely due to the averaging of Zα1 and Zα2 induced changes; however, Zα1 seems to prefer the already stabilized Z-form, mirroring the ^{15}N-HSQC analyses.

In contrast to the m^8G4 imino signal intensities, the addition of $Z\alpha^{ADAR1}$, $Z\alpha1^{ZBP1}$, $Z\alpha2^{ZBP1}$, or Tandem^{ZBP1} resulted in a relative decrease of the signal intensities of imino resonances for the d(CpG)$_3$, r(CpG)$_3$, and LNA (CpG)$_3$ oligos (Supplementary Figure S15B-D). The oligos in the B-form, A-form, and A-like conformations, respectively, are relatively stable under these experimental conditions and do not adopt appreciable Z-form populations or melted intermediates in the absence of protein, even at 42°C [8]. The decrease in imino proton signals could reflect increased exchange with water as a result of active right-to-left-handed conversion processes in the presence of the ZBDs or increased R2 relaxation as a result of increased complex size or increased μs-ms exchange contributions due to active conversion processes. Similar to previous reports [19], titration of $Z\alpha^{ADAR1}$ into the d(CpG)$_3$ oligo resulted in the gradual separation of the B-form G2 and G4 imino resonances into new Z-form positions at ~13.20 and ~13.05 ppm, respectively (Supplementary Figure S15B). Interestingly, G4, but not G2, imino resonances in the $Z\alpha1^{ZBP1}$–d(CpG)$_3$ titration series shifted to a position similar to that observed in the $Z\alpha^{ADAR1}$ series. Instead, the G2 resonance gradually broadened as [P]/[NA] ratios were increased. Titration of $Z\alpha2^{ZBP1}$ with the d(CpG)$_3$ oligo, in contrast, did result in the appearance of two unique Z-form G2 and G4 resonances at high [P]/[NA] ratios. Tandem^{ZBP1} largely behaved similarly to $Z\alpha2^{ZBP1}$, however, analysis was hampered due to heavy line broadening, likely because of increased molecular size. The increased ability of $Z\alpha2^{ZBP1}$ and Tandem^{ZBP1} to induce a more stable Z-conformation G2 resonance than $Z\alpha1^{ZBP1}$ reflects the CD results that showed that $Z\alpha2^{ZBP1}$ was more efficient in stabilizing the Z-conformation. Furthermore, compared to $Z\alpha1^{ZBP1}$ and $Z\alpha2^{ZBP1}$, the imino resonances of the $Z\alpha^{ADAR1}$ – d(CpG)$_3$ titration series were significantly more broadened, indicating more substantial contributions from intermediate exchange, mirroring the results of the 2D ^{15}N-HSQC analyses.

Likewise, the exchange regimes of the imino resonances for the 1D $Z\alpha^{ADAR1}$ – r(CpG)$_3$ titration series matched the slow exchange regime seen in the ^{15}N-HSQCs. Peak positions for the B-form G2 and G4 resonances gradually disappeared and reappeared in the Z-form positions at ~13.2 and ~13.0 ppm, respectively, as relative [P]/[NA] ratios were increased and closely matched the final peak positions of other studies (Supplementary Figure S15C, [19]). Similarly, exchange regimes for the 1D titration series of $Z\alpha1^{ZBP1}$, $Z\alpha2^{ZBP1}$, and Tandem^{ZBP1} with the r(CpG)$_3$ oligo matched the ^{15}N-HSQC series and occurred almost exclusively on the fast time scale with relatively minor signal loss, with the exception of Tandem^{ZBP1}, which experienced larger signal losses. Additionally, while imino resonances for the $Z\alpha^{ADAR1}$ series resulted in downfield shifts, the ZBP1 constructs displayed different final peak positions for the RNA 6-mer imino resonances, confirming a different binding mode and lack of converting ability for the unmodified r(CpG)$_3$ oligo. Imino shifts for the LNA (CpG)$_3$ oligo also largely match the trends seen in the ^{15}N-HSQCs (Supplementary Figure S15D). G2 and G4 imino resonances gradually shift throughout the titration series, indicating fast on/off rates, however, unlike every other 1D titration series, largest CSPs occurred at low relative [P]/[N] ratios and gradually shifted back to the positions of free oligo at high relative [P]/[N] ratios. In line with previous reports [18], imino resonances were also heavily broadened at low relative [P]/[N] ratios, and with the multimodal binding behaviors seen in the 2D ^{15}N-HSQC spectra indicate that multiple LNA (CpG)$_3$ oligos are capable of binding the same $Z\alpha^{ADAR1}$ protein. Interestingly, $Z\alpha1^{ZBP1}$ and $Z\alpha2^{ZBP1}$ show slightly different behaviors in the 1D series, with the G4 imino resonance shifting linearly as relative [P]/[N] ratios increase, whereas the G2 imino resonance has the largest CSP at low relative [P]/[N] ratios and shift back towards free oligo at high relative [P]/[N] ratios. Unlike $Z\alpha^{ADAR1}$, where signal intensities increased as relative protein concentrations increased, increasing the relative concentrations of both $Z\alpha1^{ZBP1}$ and $Z\alpha2^{ZBP1}$ caused a decrease in the height of the imino resonances, suggesting that both domains do not have multiple binding sites for LNA oligos.

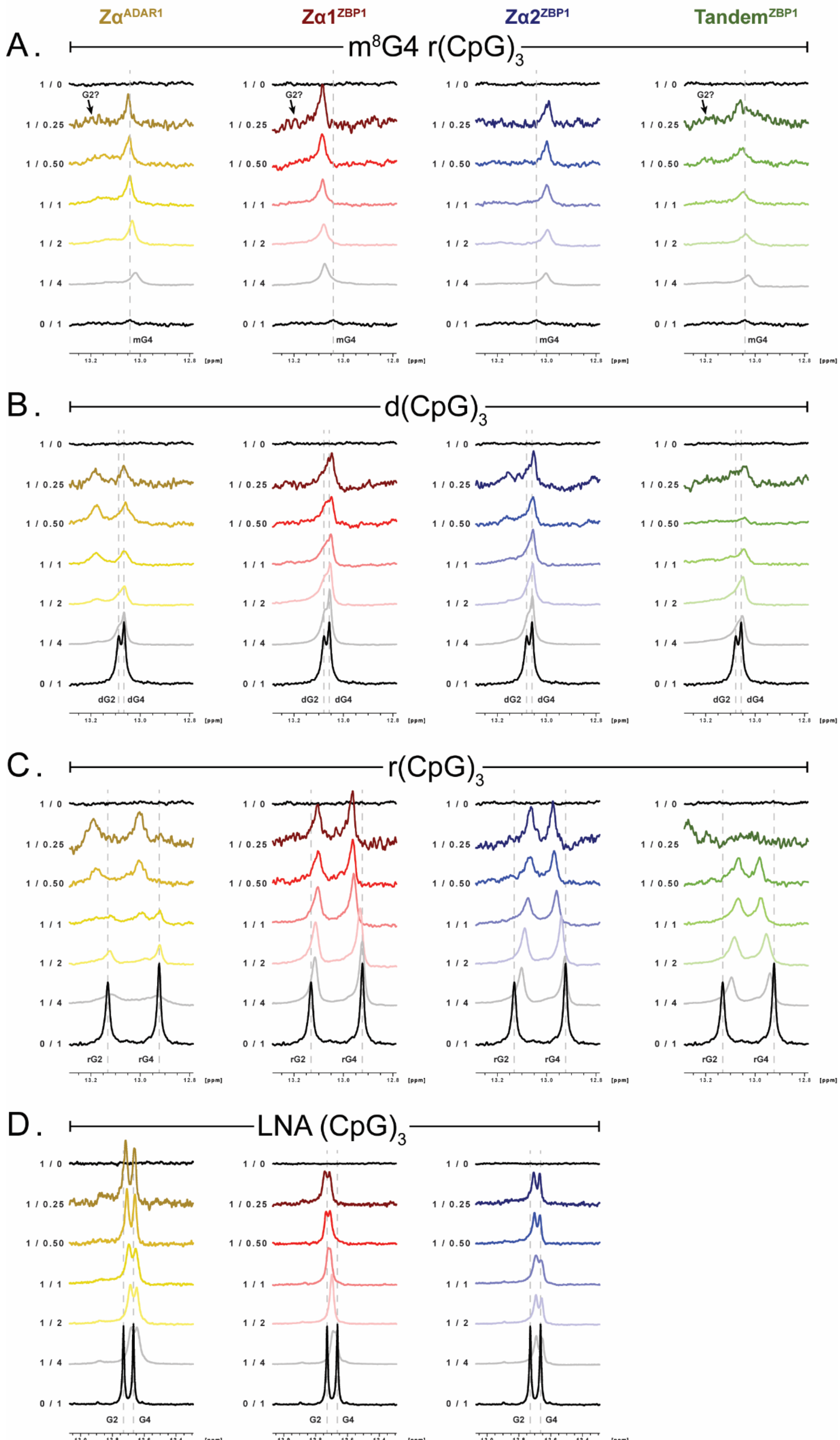

Zα^ADAR1
Zα1^ZBP1
Zα2^ZBP1
Tandem^ZBP1
A.
m8G4 r(CpG)3
B.
d(CpG)3
C.
r(CpG)3
D.
LNA (CpG)3
1 / 0
1 / 0.25
1 / 0.50
1 / 1
1 / 2
1 / 4
0 / 1
G2?
mG4
dG2
dG4
rG2
rG4
G2
G4
[ppm]

Supplementary Figure S15. 1D imino spectra of different 6-mer oligos upon titration of $Z\alpha^{ADAR1}$ (yellow), $Z\alpha1^{ZBP1}$ (red), $Z\alpha2^{ZBP1}$ (blue), and $Tandem^{ZBP1}$ (green) measured at 35°C. Each spectrum was artificially scaled up/down to account for differences in nucleic acid concentrations and protein/nucleic acid ratios are listed to the left of each spectrum. Grey dashes represent the starting positions of the resonances in the free spectra. **(A)** 1D imino titration series of ZBD constructs with the m^8G4 $r(CpG)_3$ oligo. The resonances of the Z-form oligo are labeled as mG4. **(B)** 1D imino titration series of ZBD constructs with the $d(CpG)_3$ oligo. The resonances of the B-form oligo are labeled as dG2 and dG4. **(C)** 1D imino titration series of ZBD constructs with the $r(CpG)_3$ oligo. The resonances of the A-form oligo are labeled as rG2 and rG4. **(D)** 1D imino titration series of ZBD constructs with the LNA $(CpG)_3$ oligo. The resonances of the A-like oligo are labeled as G2 and G4.

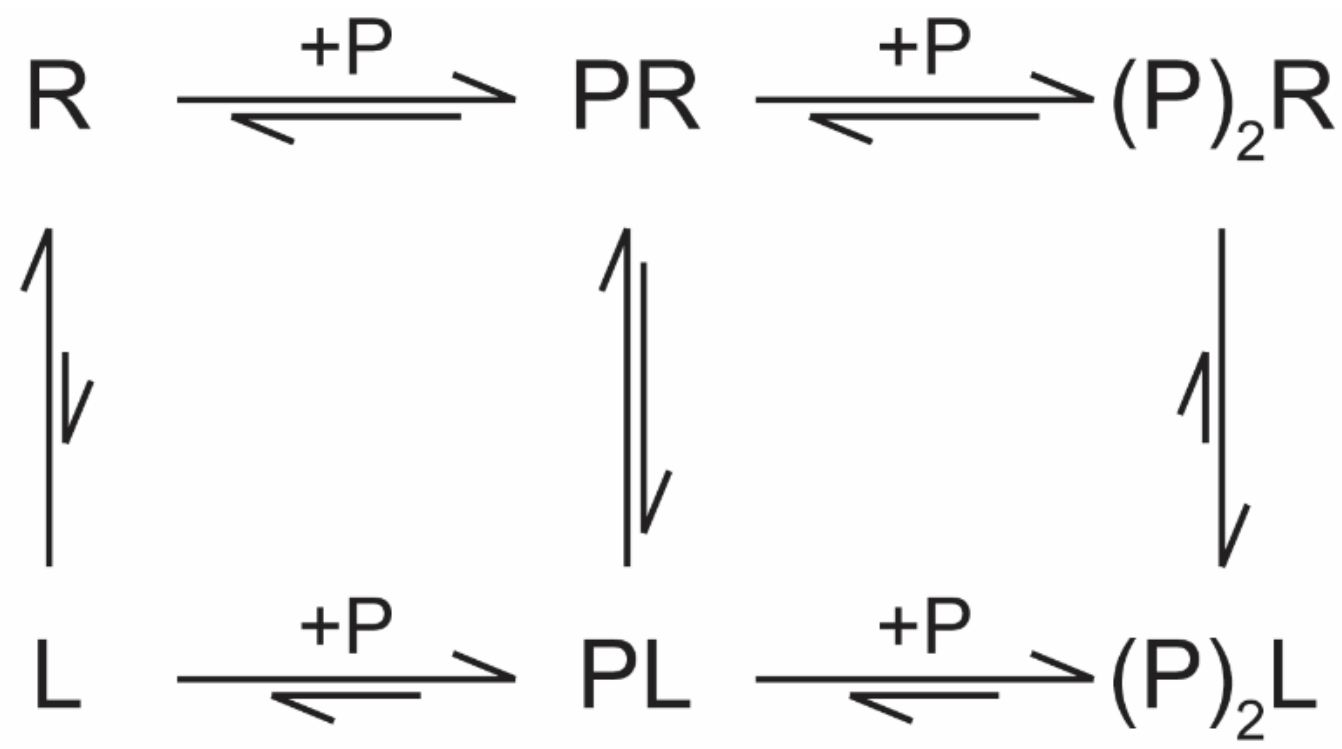

Supplementary Figure S16. Induced fit sequential binding model describing the binding of ZBDs (denoted as P) to nucleic acids including conformational changes of right-handed (R) duplexes to the left-handed (L) Z-conformation. Equilibria arrows are representative of relative population dynamics and favorabilities but do not represent measured values.

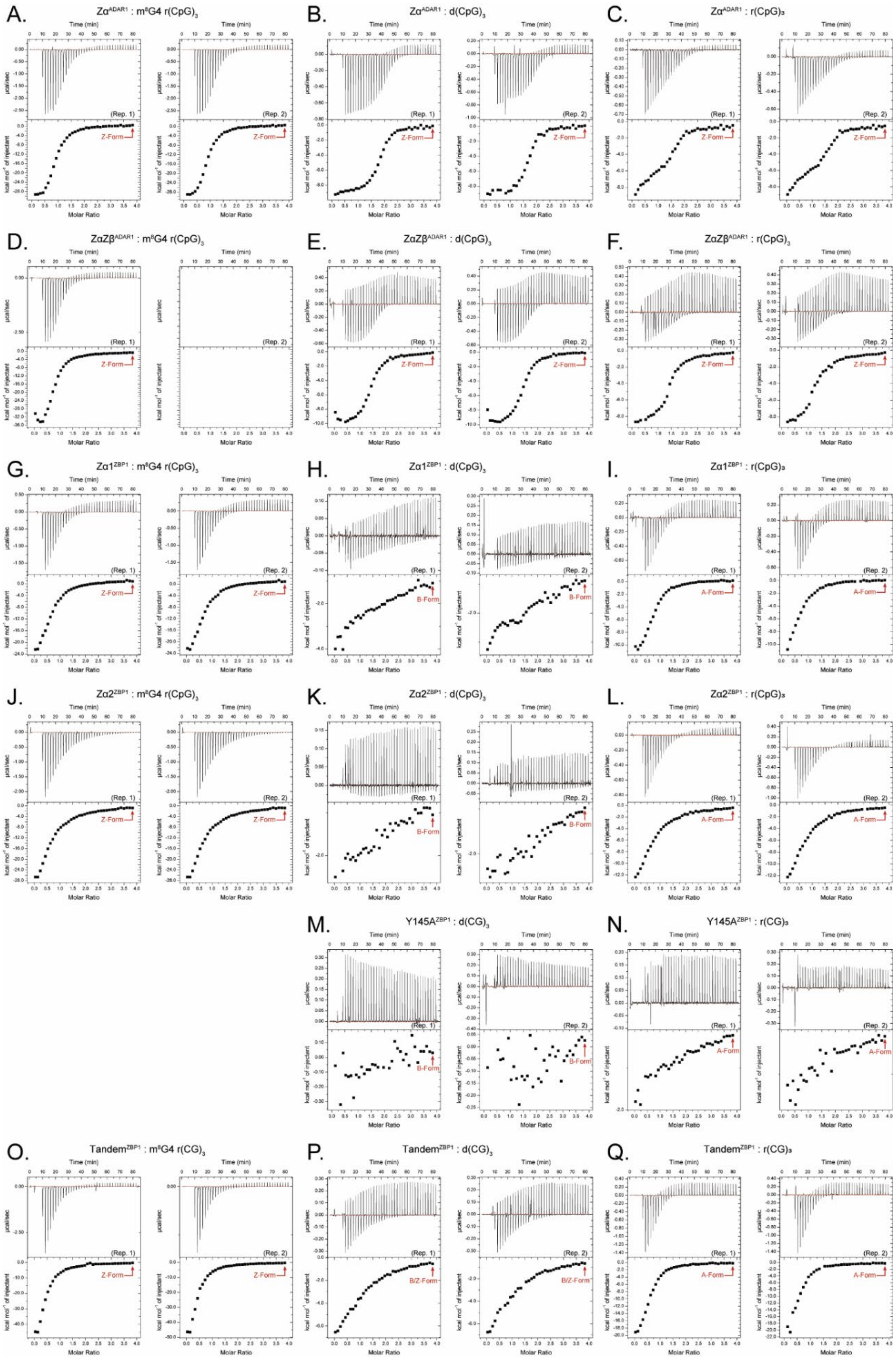

A.
Zα^ADAR1 : m⁸G4 r(CpG)3
B.
Zα^ADAR1 : d(CpG)3
C.
Zα^ADAR1 : r(CpG)3
D.
ZαZβ^ADAR1 : m⁸G4 r(CpG)3
E.
ZαZβ^ADAR1 : d(CpG)3
F.
ZαZβ^ADAR1 : r(CpG)3
G.
Zα1^ZBP1 : m⁸G4 r(CpG)3
H.
Zα1^ZBP1 : d(CpG)3
I.
Zα1^ZBP1 : r(CpG)3
J.
Zα2^ZBP1 : m⁸G4 r(CpG)3
K.
Zα2^ZBP1 : d(CpG)3
L.
Zα2^ZBP1 : r(CpG)3
M.
Y145A^ZBP1 : d(CG)3
N.
Y145A^ZBP1 : r(CG)3
O.
Tandem^ZBP1 : m⁸G4 r(CG)3
P.
Tandem^ZBP1 : d(CG)3
Q.
Tandem^ZBP1 : r(CG)3
Time (min)
µcal/sec
kcal mol⁻¹ of injectant
Molar Ratio
(Rep. 1)
(Rep. 2)
Z-Form
B-Form
A-Form
B/Z-Form

Supplementary Figure S17. Raw Isothermal Titration Calorimetry measurements of Zα^{ADAR1} **(A-C),** ZαZβ^{ADAR1} **(D-F)**, Zα1^{ZBP1} **(G-I)**, Zα2^{ZBP1} **(J-L)**, and Tandem^{ZBP1} **(M-O)** into m^{8}G4 r(CpG)$_3$, d(CpG)$_3$, and r(CpG)$_3$ oligos, respectively. Y145A^{ZBP1} **(P,Q)** was titrated into d(CpG)$_3$ and r(CpG)$_3$ oligos, respectively. All measurements were done in duplicate and measured at 35°C with 2 min injection intervals.

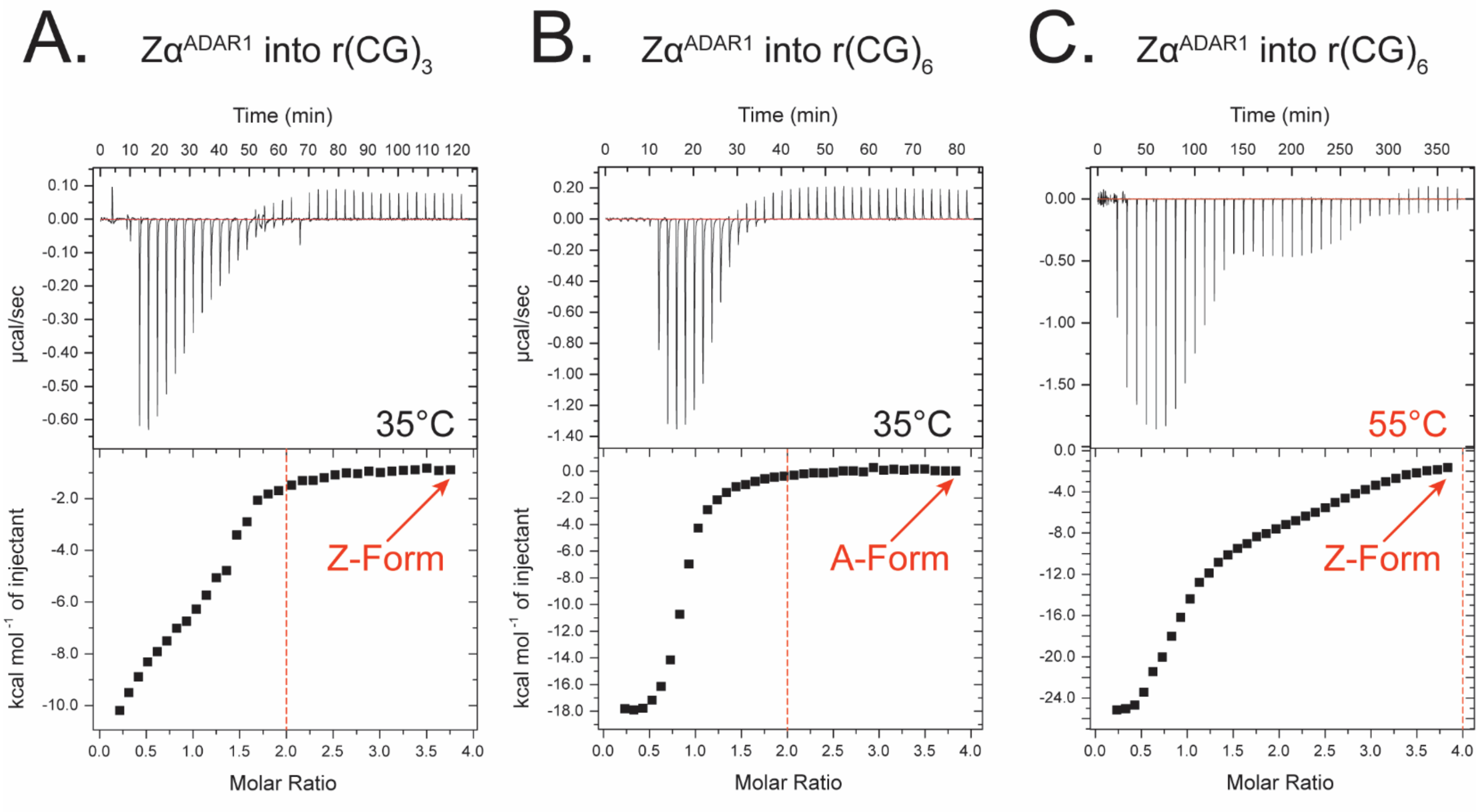

Supplementary Figure S18. Raw Isothermal Titration Calorimetry measurements comparing differences in stoichiometry between a 6-mer r(CpG)$_3$ oligo at 35°C and 2 min injection intervals **(A)**, a 12-mer r(CpG)$_6$ oligo at 35°C and 2 min injection intervals **(B)**, and the same 12-mer r(CpG)$_6$ oligo at 55°C and 10 min injection intervals **(C)**.

REFERENCES

1. Luscombe, N.M., R.A. Laskowski, and J.M. Thornton, *NUCPLOT: a program to generate schematic diagrams of protein-nucleic acid interactions.* Nucleic acids research, 1997. **25**(24): p. 4940–4945.
2. Schwartz, T., et al., *Crystal structure of the Zα domain of the human editing enzyme ADAR1 bound to left-handed Z-DNA*. Science, 1999. **284**(5421): p. 1841–1845.
3. Placido, D., et al., *A left-handed RNA double helix bound by the Zα domain of the RNA-editing enzyme ADAR1*. Structure, 2007. **15**(4): p. 395–404.
4. He, J., et al., *ZBP1 senses spliceosome stress through Z-RNA: DNA hybrid recognition*. Molecular Cell, 2025. **85**(9): p. 1790–1805. e7.
5. Schwartz, T., et al., *Structure of the DLM-1–Z-DNA complex reveals a conserved family of Z-DNA-binding proteins.* Nature structural biology, 2001. **8**(9): p. 761–765.
6. Ha, S.C., et al., *The crystal structure of the second Z-DNA binding domain of human DAI (ZBP1) in complex with Z-DNA reveals an unusual binding mode to Z-DNA*. Proceedings of the National Academy of Sciences, 2008. **105**(52): p. 20671–20676.
7. Beck, L.G., et al., *Solution NMR backbone assignment of the N-terminal tandem Zα1-Zα2 domains of Z-DNA binding protein 1*. Biomolecular NMR assignments, 2024. **18**(2): p. 245–252.
8. Nichols, P.J., et al., *Z-form adoption of nucleic acid is a multi-step process which proceeds through a melted intermediate.* Journal of the American Chemical Society, 2023. **146**(1): p. 677–694.
9. Brown, B.A., et al., *The Zα domain of the editing enzyme dsRNA adenosine deaminase binds left-handed Z-RNA as well as Z-DNA.* Proceedings of the National Academy of Sciences, 2000. **97**(25): p. 13532–13536.
10. Krall, J.B., et al., *Structure and Formation of Z-DNA and Z-RNA*. Molecules, 2023. **28**(2): p. 843.
11. Foloppe, N. and A.D. MacKerell, *Conformational properties of the deoxyribose and ribose moieties of nucleic acids: a quantum mechanical study.* The Journal of Physical Chemistry B, 1998. **102**(34): p. 6669–6678.
12. Ghosh, A. and H.X. Zhou, *Determinants for fusion speed of biomolecular droplets.* Angewandte Chemie International Edition, 2020. **59**(47): p. 20837–20840.
13. Ruff, K.M., et al., *Advances in understanding stimulus-responsive phase behavior of intrinsically disordered protein polymers.* Journal of molecular biology, 2018. **430**(23): p. 4619–4635.
14. Quiroz, F.G. and A. Chilkoti, *Sequence heuristics to encode phase behaviour in intrinsically disordered protein polymers.* Nature materials, 2015. **14**(11): p. 1164–1171.
15. Miyahara, T., H. Nakatsuji, and H. Sugiyama, *Similarities and differences between RNA and DNA double-helical structures in circular dichroism spectroscopy: a SAC–CI study.* The Journal of Physical Chemistry A, 2016. **120**(45): p. 9008–9018.
16. Puglisi, J.D. and I. Tinoco Jr, *[22] absorbance melting curves of RNA*, in *Methods in enzymology*. 1989, Elsevier. p. 304–325.
17. Felsenfeld, G. and S.Z. Hirschman, *A neighbor-interaction analysis of the hypochromism and spectra of DNA*. Journal of Molecular Biology, 1965. **13**(2): p. 407–427.
18. Nichols, P.J., et al., *Zα Domain of ADAR1 Binds to an A-Form-like Nucleic Acid Duplex with Low Micromolar Affinity.* Biochemistry, 2024. **63**(6): p. 777–787.
19. Lee, A.-R., et al., *NMR dynamics study reveals the Zα domain of human ADAR1 associates with and dissociates from Z-RNA more slowly than Z-DNA.* ACS chemical biology, 2018. **14**(2): p. 245–255.
